# Stress-Induced Morphological, Cellular and Molecular Changes in the Brain—Lessons Learned from the Chronic Mild Stress Model of Depression

**DOI:** 10.3390/cells9041026

**Published:** 2020-04-21

**Authors:** Ahmad Raza Khan, Lili Geiger, Ove Wiborg, Boldizsár Czéh

**Affiliations:** 1 Centre of Biomedical Research, Sanjay Gandhi Post Graduate Institute (SGPGI) Campus, Lucknow-226017, U.P, India; 110ahmadkhan@gmail.com; 2 Neurobiology of Stress Research Group, Szentágothai Research Centre, University of Pécs, 7624 Pécs, Hungary; g.lilly92@gmail.com; 3 Department of Laboratory Medicine, Medical School, University of Pécs, 7624 Pécs, Hungary; 4 Department of Health Science and Technology, Aalborg University, 9220 Aalborg, Denmark; ow@hst.aau.dk

**Keywords:** animal model, CMS, depressive disorder, diffusion MRI, magnetic resonance imaging, microRNA, MRI, neuroplasticity, proton magnetic resonance spectroscopy, ^1^H MRS

## Abstract

Major depressive disorder (MDD) is a severe illness imposing an increasing social and economic burden worldwide. Numerous rodent models have been developed to investigate the pathophysiology of MDD. One of the best characterized and most widely used models is the chronic mild stress (CMS) model which was developed more than 30 years ago by Paul Willner. More than 2000 published studies used this model, mainly to assess novel compounds with potential antidepressant efficacy. Most of these studies examined the behavioral consequences of stress and concomitant drug intervention. Much fewer studies focused on the CMS-induced neurobiological changes. However, the stress-induced cellular and molecular changes are important as they may serve as potential translational biomarkers and increase our understanding of the pathophysiology of MDD. Here, we summarize current knowledge on the structural and molecular alterations in the brain that have been described using the CMS model. We discuss the latest neuroimaging and postmortem histopathological data as well as molecular changes including recent findings on microRNA levels. Different chronic stress paradigms occasionally deliver dissimilar findings, but the available experimental data provide convincing evidence that the CMS model has a high translational value. Future studies examining the neurobiological changes in the CMS model in combination with clinically effective antidepressant drug intervention will likely deliver further valuable information on the pathophysiology of MDD.

## 1. Introduction

Major depressive disorder (MDD) is a complex and potentially life threatening disorder imposing a severe social and economic burden worldwide. MDD is projected to become a leading cause of disability by the end of 2030, according to a recent World Health Organization (WHO) report [1]. There are effective treatment strategies, but many patients remain undiagnosed, or do not respond sufficiently to antidepressant therapy. A major limitation of effective therapy is that the diagnosis of MDD is still based on subjective criteria as was recently updated in the Diagnostic and Statistical Manual for mental disorders (DSM V) [2,3]. Therefore, objective biomarkers are needed to detect the pathophysiological changes specific for MDD. Despite decades of intensive research at the clinical and preclinical levels, objective biomarkers are still not available to aid the unbiased diagnosis of MDD. In vivo neuroimaging of the structural and functional changes that are present in the brains of depressed individuals may help us to develop such diagnostic markers. For example, hippocampal volume decrease has been intensively studied as a neuroimaging biomarker for MDD [4,5,6,7,8]. One should emphasize that the exact cellular pathophysiology underlying this overall hippocampal volume change is still debated [9,10,11]. Animal models of depression are valuable tools as they may improve our understanding of the underlying pathophysiology, could facilitate the identification of disease specific biomarkers, and aid the development of new antidepressant treatment strategies. Fortunately, it is now clear that animal models based on chronic stress protocols recapitulate many of the structural alterations that have been demonstrated in the brains of depressed patients, therefore such models are valuable tools to investigate the pathophysiology of the disease [12,13].

The chronic mild stress (CMS) model, which was originally developed by Paul Willner [14] is one of the best characterized and most widely used rodent models of MDD [15,16]. Originally, the model was developed for rats—and it works best with rats—but since then, many research groups adapted the protocol for mice. The CMS model is an extensively validated and realistic model of depression providing (1) high face-validity, for example, similarity between the behavioral phenotype and the clinical symptom profile; (2) predictive validity, for example, symptom amelioration by clinical effective antidepressant treatments and conversely unaffected by clinical ineffective treatment of the human disorder; (3) etiological validity, for example, triggering by events known to be important for eliciting the human disorder; and (4) construct validity, for example, similar neurobiological underpinnings [15,16,17,18]. Most importantly the CMS model, like a few related rodent models based on stress exposure, such as social defeat and learned helplessness models [13,19,20], all mimic one of the core symptoms in depression; anhedonia, which is a decreased interest in activities that used to be pleasurable in the premorbid state. In rodents, this behavior is measured as a decreased voluntary consumption of a dilute sucrose solution. The key component of the CMS protocol is that during a prolonged period rodents are exposed to a sequential number of mild and variable micro-stressors including alterations in day/night illumination, housing conditions, food/water availability and so forth [16,17].

**Figure 1:**
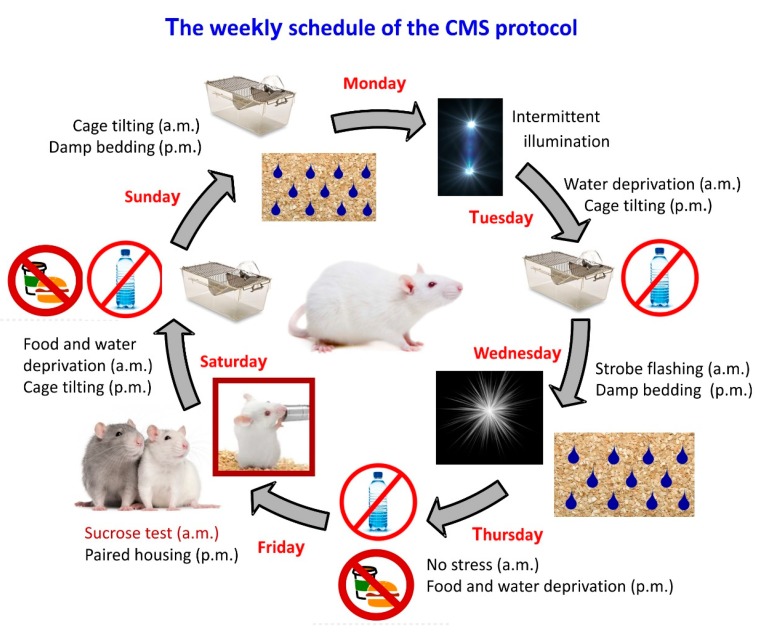
The weekly protocol of the chronic mild stress (CMS) treatment. Adult male Wistar rats are subjected every day to a different micro-stressor lasting for 10–14 h. Intermittent illumination: lights on and off every 2 h; Cage tilting into a 45° position; Strobe flashing: stroboscopic lightning; Damp bedding: pouring water into the cage to damp the beddings; Paired housing: pairing two rats by having an unfamiliar partner at each grouping session. This weekly schedule is typically repeated over a period of 4–8 weeks.

In our own studies, we have mainly used a Wistar rat strain, which is an outbred strain, and subjected the animals to a series of micro-stressors every day (Figure 1). Likely due to their genetic within-strain variability rats respond differently and individually to stress exposure; some are susceptible to stress exposure, while some are capable of actively coping with stressors, being stress resilient [21]. Stress susceptible rats show deficits in reward sensitivity, measured as a decrease in sucrose consumption, as well as impairments in a number of additional behavioral, histological and functional readouts [22,23]. These deficits may be reversed by chronic, but not acute, antidepressant treatment; however, a substantial fraction are treatment resistant and do not recover [24]. Thus, the clinical time-lag during remission, as well as the inadequate antidepressant drug efficacy, is well simulated in the CMS model and makes it a valuable tool in antidepressant drug screening, as well as for studying disease pathology and mechanisms underlying drug action, treatment resistance and stress resilience mechanisms.

In the literature of CMS experiments, most studies investigated the potential antidepressant efficacy of novel compounds by examining stress-induced behavioral changes and subsequently assessing whether concomitant drug treatment was able to reverse the stress-induced behavioral alterations. However, there are also several studies which focused on CMS-induced cellular and molecular changes. These are important as they may help us to unravel the neurobiological background of MDD. We should emphasize, that it is still debated whether the chronic-stress models are truly helpful and some scientists argue that the chronic stress-induced cellular alterations actually do not explain the neurobiological basis of depressive episodes [25]. It might also be that stress results in additional molecular or cellular alterations which have not yet been discovered, but more directly contribute to the development of depressive episodes.

The aim of the present review is to provide an up-to date summary of our current knowledge on the structural and molecular alterations that have been described specifically in the CMS model by in vivo imaging and postmortem histopathological studies. Data from the CMS model are rapidly accumulating and we believe that such a summary could aid future research. We argue that these findings are of great clinical importance as they have strong translational value. Not only because they could shed light on the neurobiology of the disease, but because they could greatly facilitate the development of objective diagnostic markers. Furthermore, different chronic stress protocols sometimes yield different results. Here we aim to list those findings that have been described exclusively with the CMS model.

The present review is structured in such a way that we discuss the key areas of stress circuitries separately and present the corresponding stress-induced cellular and molecular changes affecting these regions. This approach is based on the available knowledge that brain areas regulating the stress response are the ones which are typically affected in MDD patients as well as in animal models of depression [13,26,27,28,29,30].

## 2. Hippocampal Formation

**Figure 2:**
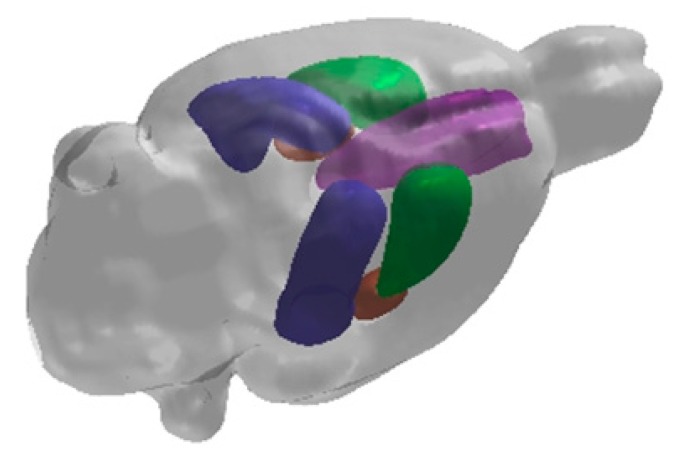
A representative 3D image of a rat brain which has been digitally reconstructed from magnetic resonance imaging (MRI) scans and depicts the medial prefrontal cortex (violet), caudate putamen (green), hippocampus (blue) and amygdala (brown).

The hippocampal formation is part of the limbic system and an important regulator of the hypothalamus-pituitary-adrenal (HPA) axis [31]. Furthermore, this brain area is highly susceptible to various insults and hippocampal volume decrease has been intensively studied as a neuroimaging biomarker for depression [4,5,6,7,8]. One important underlying cellular mechanism for this high sensitivity is the fact that hippocampal neurons have high expression levels of receptors for glucocorticoids and mineralocorticoids. These receptors mediate a variety of effects on neuronal excitability, neurochemistry, and structural plasticity [32,33]. In consequence, the chronic stress-induced surge of adrenal steroids alters cellular functions and plasticity in the hippocampus and results in dendritic atrophy, loss of dendritic spines and inhibition of neurogenesis in the adult dentate gyrus [34,35,36]. These cellular changes contribute to the gross hippocampal volume loss [9,10] since the loss of dendritic and axonal material, as well as glial changes, are the most likely contributing factors to the hippocampal volume loss. Massive neuronal loss—which has been documented in earlier studies—is no longer considered an important contributing factor [9,10,36,37,38]. Our own MRI findings, which were primarily based on regions of interest (ROIs) (Figure 2), also imply the absence of massive neuronal loss, which if present would affect the diffusion tensor imaging (DTI) based mean diffusivity (MD), fractional anisotropy (FA) parameters, and cellular markers of proton magnetic resonance spectroscopy (^1^H MRS) [28,29,30,39,40,41].

One of the most well-known stress-induced cellular change is the dendritic atrophy of pyramidal neurons which has been documented first in the CA3 area, but later also in the CA1 region [42]. There is also evidence of stress-induced dendritic atrophy of dentate granule cells [42,43]. This stress-induced dendritic reorganization is typically accompanied by reduced spine density as it was shown in hippocampal CA1 pyramidal neurons [44]. CMS reduces the length of apical dendrites [45] as well as spine density and also alters spine morphology in pyramidal neurons of the hippocampal CA3 area [46].

Another thoroughly investigated form of neuroplasticity is the generation of newborn neurons in the adult dentate gyrus. Large number of studies documented that CMS inhibits adult hippocampal neurogenesis [24,43,47,48,49,50,51,52,53,54,55,56,57,58,59]. Follow-up studies also found a significant decrease in the total number of dentate granule cells in stress exposed rats [60,61]. Furthermore, it has been shown that these cellular changes are accompanied by subcellular cytoskeletal alterations, that is, altered expression of alpha-tubulin isoforms and phospho-MAP-2 indicating impaired microtubule dynamics [62]. Numerous experimental data suggest that conditions that produce dendritic retraction make the hippocampus vulnerable to neurotoxic or metabolic insults and as the “glucocorticoid cascade hypothesis” postulates, when these conditions are long-lasting then eventually the hippocampus will shrink, and its impaired functioning will lead to disturbed HPA-axis regulation and cognitive deficits [63,64,65].

As more recent studies point out, chronic stress affects another important neuronal cell type, namely the GABAergic local circuit inhibitory neurons [66,67,68,69,70]. GABAergic interneurons of the hippocampus make up only 10–20% of the total neuronal population [71], still their remarkable anatomical and physiological diversity allows them to regulate virtually all aspects of cellular and neural circuit function [72,73]. Importantly, recent theories highlight the role of these GABAergic neurons, of the hippocampus and neocortex, in the pathogenesis of MDD [74,75,76]. These concepts are based on the observations in clinical studies involving depressed patients and chronically stressed animal models which all demonstrate reduced GABA levels in the brain, as well as decreased expression of GABAergic interneuron markers, and alterations of GABA_A_ and GABA_B_ receptor expressions in multiple brain areas. In the CMS model, we have also demonstrated stress-induced changes in the number of hippocampal interneurons [70]. We found reduced number of parvalbumin, somatostatin, calretinin and neuropeptide Y expressing neurons in specific hippocampal subareas [70]. Parvalbumin-positive GABAergic interneurons, which are crucially involved in processing complex cognitive tasks, seem to be particularly vulnerable to stress exposure [66,67,70,77,78]. In line with these observations, a significant decrease in the dendritic arborization of CA1 GABAergic interneurons were found together with a reduced expression of glutamic acid decarboxylase 67 (GAD67) in different layers of CA1 and CA3 regions of the hippocampus [68]. GAD67 is an isoform of the GAD enzyme that catalyzes the decarboxylation of glutamate to GABA. The stress-induced dysfunctional inhibitory network eventually leads to impaired rhythmic oscillations and, as a consequence, to cognitive deficits which are common in stress-related psychiatric disorders [77].

**Figure 3:**
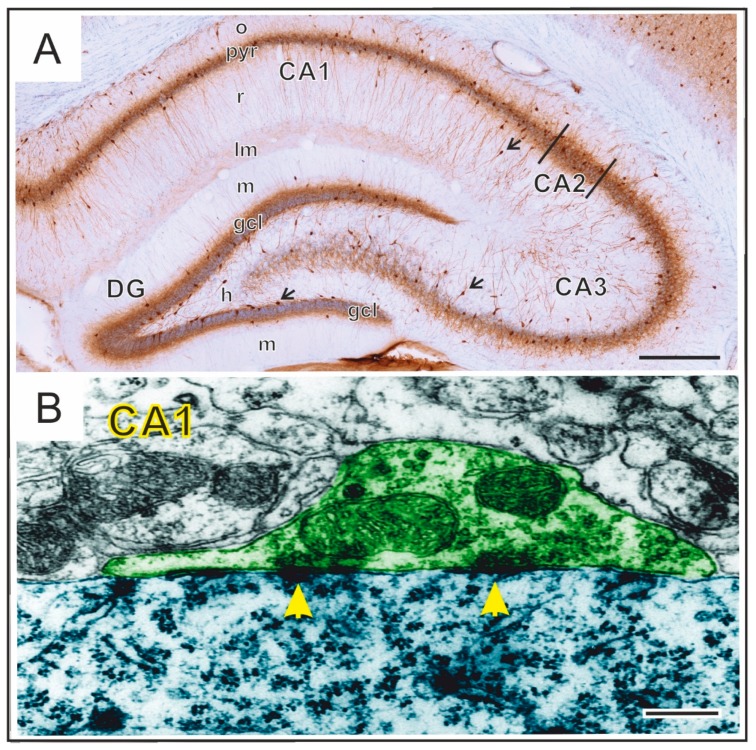
A representative microscopic image of the rat hippocampus (**A**). In this coronal section, the GABAergic perisomatic inhibitory neurons were visualized with parvalbumin-immunohistochemistry, see the numerous brown cells, some of them are indicated with arrows. Their dense axon fibers delineate the two distinct cell layers (gcl and pyr) formed by the granule cells (gcl) of the dentate gyrus and pyramidal neurons (pyr) of the CA1–3 areas. Abbreviations: o: stratum oriens; pyr: stratum pyramidale; r: stratum radiatum; lm: stratum lacunosum-moleculare; m: dentate molecular layer (stratum moleculare); gcl: granule cell layer (stratum granulosum); h: hilus proper; DG: dentate gyrus; CA1–3: Cornu Ammonis 1–3. A representative electron microscopy image of perisomatic contacts and inhibitory synapses in the CA1 area (**B**). The axon terminal (green) of an inhibitory neuron projects to the soma (blue) of a pyramidal cell. Synaptic densities are indicated with yellow arrowheads. Scale bars: 0.5 mm on **A** and 250 nm on **B**.

We have also carried out a detailed electron microscopic analysis to quantify the frequency and morphology of perisomatic inhibitory synapses in the hippocampal CA1 area (Figure 3). Surprisingly, the number of these inhibitory synapses was not affected by stress [79]. This finding was unexpected since (1) we found a reduced number of parisomatic GABAergic inhibitory neurons in the same animals [79]; and (2) there are numerous reports on the remarkable remodeling of the excitatory synapses on spines that has been reported in response to stress and depressive-like behavior [80,81,82,83]. Not only changes in synapse numbers have been documented, but stress can result in a significant remodeling of the ultrastructural morphology of individual excitatory synapses. Specifically, stress-exposure reduced the length of the synaptic active zones in the dentate gyrus and reduced the thickness of the postsynaptic density in the CA1 area [84]. Another quantitative electron microscopy study reported that, in animals exposed to CMS, the density of synapses and small dendritic spines were increased in the inner and outer molecular layers of the dentate gyrus [85] which all suggests a significant remodeling within the hippocampal neuronal circuitry.

The stress-induced morphological changes of the GABAergic network are complemented by in vitro electrophysiological findings, which document dysfunctional GABAergic neurotransmission in the hippocampi of chronically stressed rats [77,86,87,88]. In addition to that, an in vivo electrophysiological study focusing on network functions documented impaired long term potentiation (LTP) induction and decreased basal synaptic transmission at the hippocampal CA3-CA1 synapses, and this was accompanied by decreased density of dendritic spines in CA1 and CA3 pyramidal neurons [89].

The more detailed neuroanatomical and functional studies document region-specific stress-induced differences in the dorsal and ventral hippocampus [45]. Specifically, the dorsal hippocampus mediates spatial navigation while the ventral hippocampus is considered to be responsible for mainly emotional and anxious behavior [90,91]. The ventral hippocampus is a potent regulator of the neuroendocrine system by inhibiting the activity of the HPA-axis and consequently diminishing glucocorticoid release from the adrenal glands [92]. Neylan et al., 2003, reported that cortisol levels are positively correlated with hippocampal *N*-acetylaspartate (NAA) levels. Partly in accordance with this, we found a significant increase in NAA levels, but only after eight weeks of CMS [30,41]. A related study reported significant increase in *N*-acetyl aspartyl glutamate (NAAG) and glutamate in the brains of CMS exposed rats [93], which suggests extracellular glutamate accumulation in the hippocampus after an increase of glucocorticoid levels [94]. Another CMS study also documented positive correlations of corticosterone with GABA/glutamine ratios and glutamate/glutamine ratios in the hippocampus in stress-exposed rats [95]. The chronic stress-induced elevation of extracellular glutamate may lead to excitotoxicity and eventually to neuronal death as proposed by the “neurotoxicity hypothesis of depression” [96,97].

As recent findings indicate, stress affects not only neurons, but also glial cells, astrocytes [98] and oligodednrocytes [99], as well as microglial cells [100]. These findings are important since abnormalities of these glial cells have been described also in the brains of depressed patients [101]. Several CMS studies reported on a robust decrease in the number of GFAP-expressing astroglia in the dentate gyrus [102,103], whereas microglial activation was detected both in vivo (with PET) and postmortem (with immunofluorescence staining, and western blotting) [104]. Furthermore, CMS exposure results in an increased number of activated microglial cells (Iba-1+) [105]. These findings have led to “gliocentric theories of depression,” in which glial cells account for the pathophysiology of depression [106,107,108,109].

Molecular factors have been studied as well, and dysregulation of neurotrophic factors, primary brain-derived neurotrophic factor (BDNF), have been pointed out as a key molecule in stress related cellular changes. It has been shown that stress reduces BDNF expression [110] as well as dendritic trafficking of BDNF and that may contribute to the stress-induced dendritic atrophy [111]. Increased expression of BDNF, or direct infusion into the hippocampus can restore dendritic atrophy which suggests that BDNF is a potent regulator of the dendritic complexity [112,113]. It has also been demonstrated that chronic treatment with antidepressant drugs stimulates BDNF-mediated signaling [114]. Notably, there is a “BDNF theory of depression” which describes BDNF as the key molecule accounting for the pathological changes underlying the symptoms of depression [115,116,117].

Finally, we should point out that recent progress in human-induced pluripotent stem cell (iPSC) technologies provide excellent novel platforms for modeling the genetic mechanisms underlying MDD [118]. These models could be useful especially for studying individual differences and for developing personalized therapies [119]. Furthermore, single-cell models allow the quantitative analysis of synaptic transmission and the plasticity of different cell lines (i.e., GABA- or glutamatergic neurons) and these models are also valuable for compound screening [120].

## 3. Prefrontal Cortex (PFC)

The prefrontal cortex (PFC) has extensive connections to other neocortical and sub-cortical brain regions and therefore has a vital role in the regulation of executive functions, emotions and memories [121]. Furthermore, the PFC also participates in the regulation of the HPA-axis [122]. Numerous studies have documented disrupted prefrontal activity and structural atrophy in depressed patients [123,124,125], and atrophy in the medical PFC has been shown to correlate with executive dysfunctions [126]. It is well documented that chronic stress can reduce the volume of the PFC both in humans and rodents [127,128,129,130]. The cellular basis of this volume reduction is most likely the loss of dendritic material, but stress-induced decrease in glial cell proliferation may also contribute to this [9,131,132]. It has also been shown that CMS promotes apoptosis in the neocortex [133]. A pioneering study documented CMS-induced apical dendritic atrophy of pyramidal neurons in the prelimbic (PrL) and infralimbic (IL) cortices together with reduced neuronal density of layer II in PrL and IL as well as a reduced volume of layer I/II in PrL and IL cortices [134]. Another study reported reduced dendritic length of PFC neurons together with dendritic spine loss and altered spine morphology in CMS treated rats [46,135]. Furthermore, in a three week paradigm of CMS, a reduced spine number in layer V pyramidal neurons was found [136]. A substantial reduction in the apical dendritic branches might be a causal factor for the significant increase in the axial kurtosis tensor that we could detect in the PFC [30,137], however, histological validation of kurtosis metrices are not possible. Furthermore, a proton magnetic resonance spectroscopy (MRS) study found a significant decrease of glutamate (Glu), glutamine (Gln), NAA + NAAG, Glx and GABA levels in the PFC of CMS animals [138].

As mentioned earlier, current theories highlight the importance of GABAergic inhibitory neurons of the neocortex in the development of depressive disorders. Dysfunctional GABAergic neurotransmission has been reported both in depressed patients and in animal models [76,139,140,141]. So far there have been some controversies in the literature, as both enhanced [142] and impaired [23] inhibition has been demonstrated in the PFC of chronically stressed animals. But it is generally accepted that chronic stress directly affects various subtypes of GABAergic interneurons in the medial PFC either by reducing the number of specific GABAergic cell types [23] or by reducing the expression of the GABA producing enzyme GAD67 [69,143]. There is also evidence that stress can increase the activity of parvalbumin-positive cells which has mainly been shown in female animals [144,145,146]. The stress-induced dysfunctions of the PFC GABAergic networks disrupt excitatory/inhibitory balance which in turn leads to disturbed emotional and cognitive functioning, as well as disrupted HPA-axis regulation [140,141,142,147].

So far, only a handful of studies examined the stress-induced ultrastructural changes in the PFC. Our group found that CMS reduced the number of excitatory synapses as well as the number of myelinated axons in the IL, while synapse membrane lengths were increased [130]. Another research group also found a significant remodeling of excitatory synapses, that is, stress reduced the length of the synaptic active zones in the anterior-cingulate (Cg1) cortex and reduced the thickness of the postsynaptic density in the PrL [84]. Yet another group reported on increased inhibitory terminals and synapses onto glutamatergic cells [142]. These findings are important since synapse loss has been documented in the PFC of depressed patients [148,149] and synaptic dysfunctions have been proposed as key factors contributing to emotional disturbances [140].

Glial cells in the PFC are highly important for inducing depressive behavior which was supported by the finding that glial loss alone may cause depressive like behavior [150]. In addition, numerous studies have demonstrated that chronic stress can alter the number of glial cells and impair their functions [105,151,152,153,154,155,156,157,158]. Furthermore, Rajkowska and co-workers reported a significant decrease in neuronal and glial cell densities in the PFC of depressed patients [159,160]. In some cases, the investigators could not differentiate between the different types of glial cells, for example, in the case of the human postmortem studies, where the samples are stained with Nissl staining. Nissl staining enables the differentiation only between neurons and glia. But there are additional studies which documented that stress affects all types of glial cells in the PFC, that is, astrocytes [154,161] and oligodednrocytes [162] as well as microglial cells [152,153]. These findings are important since abnormalities of these glial cells have also been described in the brains of depressed patients [101]. These findings have led to “gliocentric theories of depression,” in which glial cells account for the pathophysiology of depression [106,107,108,109].

## 4. Other Neocortical Areas

Most of the clinical and preclinical research on depression has focused on stress-circuitries of the brain but recent studies have shown that the cerebral cortex is not merely a conduit to sub-cortical regions of the brain but has a significant role in the etiology and therapy of mood disorders [163]. For example, psychomotor retardation is a key component of depression which has a significant negative impact on the overall functioning of depressed patients [164]. Among others, a slumped posture, and decreased or slowed movements of hands, legs, torso and head are typical symptoms. As a potential contributing factor, a clinical DTI study reported microstructural alterations of cortico-cortical white matter motor pathways which was also related to psychomotor retardation of depressed patients [165]. Another MRI study reported decreased cerebral blood flow in the primary motor cortex in MDD patients with psychomotor retardation [166] and a recent meta-analysis of whole-brain studies revealed reduced gray matter volumes of the right supplementary motor area alterations in first episodes of depression [167]. Functional and neuroanatomical disturbances have also been found in the auditory cortex of MDD patients [168,169]. Further MRI studies documented significant thinning of several cortical areas, as well as widespread reduction of gray matter areas have been documented [170,171].

In our pre-clinical studies we also observed significant microstructural alterations in the motor and auditory cortical regions in the CMS model using diffusion MRI and immunohistochemistry [28]. We found a significant decrease in extracellular diffusivity in the auditory cortex of CMS exposed rats, presumably due to the moderate increase of neurite density level [28]. In support of this, an activated auditory cortex was reported in rats after CMS exposure [67]. Another longitudinal structural MRI study also revealed significant alterations in auditory, motor and somatosensory cortex [172]. These structural findings were associated with increased functional connectivity in the network [172]. These data suggest an as yet unexplained role of the auditory cortex in mood disorders. Notably, available evidence documents that the basolateral amygdala is essential to the development of plasticity in the auditory thalamus to support activation of auditory cortex during fear conditioning [173]. We also observed a significant thinning of the motor cortex in stress-exposed rats [28] and another study reported increased apoptosis in the neocortex of CMS treated rats [133]. These structural findings were associated with increased functional connectivity in the network [172]. A global gray matter atrophy in the brains of depressed patients [174,175] also highlight the importance of other cortical areas in the pathogenesis of MDD.

## 5. Amygdala

The amygdala is part of the limbic system and is crucially involved in the regulation of the stress response and mediates the influence of stress on memory consolidation and recall [176]. The amygdala is a key brain area in emotional reactivity and therefore disturbed functioning of the amygdala has been documented in depressed individuals [177] and unmedicated depressed individuals have reduced amygdala volume [178]. In general, it is accepted that the amygdala is hyperactive in depressed patients [179].

Corresponding to this sustained hyperactivity, chronic stress models repeatedly document increased dendritic length and complexity in the amygdala [180]. We also found a significant increase in neurite density based on biophysical modeling of diffusion MRI data in the amygdala of CMS-exposed animals [39,40]. A follow-up study demonstrated that the recovery of the microstructural alterations in the amygdaloid complex are delayed in comparison to hippocampal recovery [181]. However, we could not substantiate these findings when we did a longitudinal CMS recovery study using diffusion MRI and in vivo ^1^H MRS [30,41]. Notably, the amygdala contributes to the long-term storage of emotionally arousing and fear-related memories after stress exposure, whereas the hippocampus and PFC are associated with stress-induced impairment of memory retrieval and working memory [176]. The significant dendritic and concomitant synaptic atrophy in the hippocampus and hypertrophy in the amygdala might be an important underpinning of this contrasting memory pattern [113]. Most likely this dendritic and synaptic hypertrophy in the amygdala represents the neurobiological basis of the hyperactivation of the amygdala, which correlates with the severity of post-traumatic stress disorders [182].

Functional studies indicate a transient increase in the excitatory neurotransmitter glutamate after acute stress exposure, which also corroborate the finding of hyperactivation of the amygdala [183]. At the same time, chronic elevation of extracellular glutamate during chronic stress results in neuronal damage and glial activation [184,185]. Furthermore, a significant decrease in the number of astrocytes has been reported in the amygdala of a patient with MDD [160] and a moderate decrease in the CMS model [39]. Contrary to the hippocampus, BDNF expression is increased in the amygdala after chronic stress exposure [114].

A quantitative electron microscopy analysis found that chronic stress results in an increased maximal thickness of the postsynaptic density in the basolateral amygdala [84]. This microstructural hypertrophy may also contribute to the significant increase in the volume of the amygdala after chronic stress exposure [186]. Notably, the volume increase of the amygdala is not a consistent finding and may be due to the CMS-induced cellular and sub-cellular plasticity, which contribute to either adaptive anxiety-related behavior or dysregulated anxiety-related behavior [187].

Finally, we should emphasize that a phylogenetically conserved “molecular signature” of depression has been demonstrated in the amygdala, as a similar pattern of altered gene expression was identified in the amygdala of MDD patients and CMS-exposed animals [188]. In this study, a large-scale gene expression was monitored in postmortem tissue from the anterior cingulate cortex and amygdala of MDD patients and the findings were then compared with data from a CMS study and confirmed by quantitative polymerase chain reaction (qPCR) and Western blot. The detected genes belonged to existing cohesive networks which indicated decreased oligodendrocyte and up-regulated neuronal structure and functions. For example genes regulating neuronal molecular pathology of principal pyramidal cells of the amygdala were detected, that is, the convergence of increased ARHGAP6 (a RhoA inhibitor), CACNB2 (voltage-dependent calcium channel), and modulators of glutamatergic synaptic plasticity (CAMK2D, EGR1), coupled with increased components of cell-matrix remodeling (MATN2, CDH13, and CHSY1), suggested increased structural and functional dendritic/synaptic compartments [188].

Others found that the stress-induced anhedonic behavior was associated with disturbed diurnal oscillation of the expression of clock genes in the mouse basolateral amygdala [189].

## 6. The Dorsal Striatum

A meta-analytic study found a significantly decreased volume of several subcortical structures including the caudate nucleus and putamen in depressed patients [190,191], which has been supported by postmortem studies reporting reduced neuronal density in the caudate nucleus of depressed patients [192]. Impaired functioning of the striatum has also been documented which was correlated with the psychomotor retardation [193].

So far, relatively little is known about the stress-induced changes in the striatum, but our findings indicate a marked reduction in neurite density and a long lasting reduction of axonal density [29]. Another in-vivo diffusion MRI study reported a stress-induced increase in axial diffusivity (AD) and a reduction of the mean kurtosis parameter compared to stress-resilient animals [194]. Another diffusion MRI based study found increased radial diffusivity (RD) in the left striatum after CMS exposure [195]. Increased RD is considered a marker of demyelination and suggests neuronal atrophy. In line with this a postmortem neuronal tracing study found differential dendritic atrophy and hypertrophy in the dorso-lateral-striatum and dorso-medial-striatum, respectively [134].

## 7. White Matter Regions

Microstructural changes of the main commissural white matter pathways, such as the corpus callosum, have been extensively documented in depressed patients [196]. Anhedonia is a core symptom of MDD and a clinical DTI study revealed a positive correlation between anhedonia and reduced FA and increased RD values in white matter pathways that connect regions critical for value encoding, representing stimulus-reward associations, and guiding value-based action selection [197]. In another DTI study, anhedonia was linked to decreased FA in the cingulum and medial forebrain bundle, a pathway that connects the ventral tegmental area and nucleus accumbens [198].

**Figure 4:**
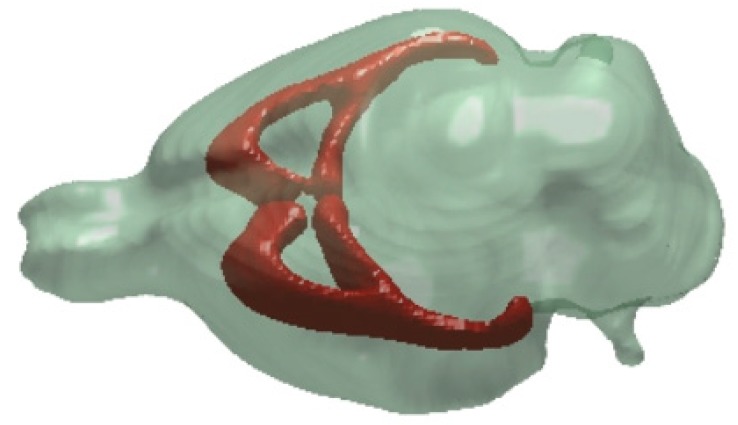
A representative 3D image showing the main commissural white matter pathways (brown) of the rat brain.

White matter alterations have been also studied in the CMS model (Figure 4). A preclinical DTI study showed decreased FA and increased mean diffusivity and radial diffusivity in the corpus-callosum of rats subjected to six weeks of CMS exposure [195]. In a clinical DTI study an increased MD of cortico-cortical white matter motor pathways was revealed [165]. This study also found a negative association of MD value with motor activity in MDD patients [165]. Another recent study documented lower FA values in the genu and frontal portion of the body of the corpus callosum in the MDD patients [199]. This study also employed white-matter tract indices (WMTI) [200,201], which revealed significantly smaller values of intra-neurite signal fraction in the body of the callosum and greater fiber dispersion in the genu [199]. Such biophysical model-based parameters have great potential for capturing subtle alterations in the tissue microstructures and may be used as a biomarker for disease onset or progression/ relapse.

## 8. Other Brain Areas

Numerous other brain areas contribute to the clinical symptoms of depressed patients and we list here a handful of studies which have investigated neurobiological changes in the CMS models.

*Habenula*: The habenula is a small, evolutionarily conserved brain structure located in the epithalamus which plays a central role in aversive processing and is hypothesized to be hyperactive in depressed patients mediating anhedonia, the central symptom of the disease. Disrupted functioning and altered volume of the habenula have been described in depressed patients [202,203]. A large scale, mass spectrometry-based, quantitative proteomic screening study found that expression of the β form of calcium/calmodulin-dependent protein kinase type II (βCaMKII) was significantly up-regulated in the lateral habenula of rats which were subjected to the learned helplessness model, or were selectively bred for the phenotype of learned helplessness (valid animal models of depression) and thus, identified βCaMKII as a powerful regulator of neuron function in the habenula and a key molecular player of depression [204].

We also did a molecular profiling study of the lateral habenula using rat whole genome expression chips (Affymetrix) and real-time quantitative polymerase chain reaction (RT-PCR) to verify the microarray results [205]. We found that stress affected intracellular cascades like growth factor receptor signaling, G-protein-coupled receptor signaling, and Wnt signaling-processes involved in the neuroplastic changes observed during the progression of depression [205]. A more recent study reported that the CMS-induced depressive-like behaviors was associated with DNA hypomethylation in the lateral habenula [206]. In this study, DNA hypomethylation increased the transcription of βCaMKII and glutamate receptor 1 in the lateral habenula and attenuated serotonin levels in the dorsal raphe nuclei. These data suggest that DNA methylation in the lateral habenula can control 5-HT neuronal activity in the dorsal raphe and by that regulates the emotional state [206]. Another research group found significant up-regulation of the somatostatin-2 receptor in the medial habenula of stress-susceptible rats [207]. Disturbed expression of the neuropeptide somatostatin has already been described decades ago in depression and other neurological disorders with mood disturbances [208,209,210].

*Nucleus accumbens*: The nucleus accumbens is located in the ventral striatum and is a key element of the mesocorticolimbic system, a brain circuit mediating reward and motivation [211]. Functional MRI studies reveal significantly weaker responses to positive reward in the nucleus accumbens of patients with MDD [191]. In the CMS model, it has been shown that stress-induced anhedonia was associated with the dendritic hypertrophy and increased spine density of the medium spiny neurons in the nucleus accumbens [212]. Another investigation found that the amplitude of molecular rhythms in the nucleus accumbens was amplified following CMS exposure and that this change correlated with mood-related behavior [213].

*Dorsal raphe nucleus*: The dorsal raphe has a central role in depressive disorders as it is the largest source of serotonin innervation to the forebrain. As the “monoamine theory of depression” postulates, deficient serotonergic neurotransmission is the key neurobiological factor underlying depressive symptoms [214,215,216]. However, in recent years this theory has been repeatedly questioned [217] and therefore this brain area nowadays receives relatively little experimental attention. Despite that, a recent state of the art optogenetic study demonstrated that optogenetic stimulation of the dorsal raphe nucleus could alleviate the depressive-like symptoms in CMS treated rats [218] and by that provided further support to the “monoamine theory of depression.”

*Suprachiasmatic nucleus*: The suprachiasmatic nucleus is a small group of neurons in the hypothalamus (right above the optic chiasm) and by its extensive interactions with many other brain areas it controls many aspects of the circadian rhythm. Disturbances in circadian rhythms are core features of depression as depressed patients often display altered circadian rhythms, sleep disturbances, and diurnal mood variation [219]. Circadian rhythmicity is a consequence of intracellular molecular mechanisms involving the so-called clock genes and disturbed functioning of these genes has been documented in various mental disorders including MDD [220,221]. In the CMS model the amplitude of molecular rhythms in the suprachiasmatic nucleus was reduced and this correlated directly with the mood-related behavior [213].

## 9. MicroRNA Changes in the CMS Model

MicroRNAs (miRNAs) are short (18–23 nucleotides), non-coding single-stranded RNA molecules that can regulate gene expression at the post-transcriptional level by binding to mRNAs and cause mRNA degradation or translational modification. miRNAs regulate approximately the half of the protein coding genes [222].

Micro RNAs are important regulators of numerous cellular processes in the CNS, that is, cell proliferation, differentiation and apoptosis. They also play a significant role in the development of depressive disorders [223,224,225]. Altered expression levels of miRNAs have been documented also in schizophrenia, Alzheimer, Parkinson’s disease, and bipolar disorder [226,227,228,229]. miRNAs are relatively stable compared to mRNAs, they are protected from degradation by forming complexes with proteins or inclusion in exosomes and their expression level can be cell/tissue type or stage- specific. By these properties, they appear to be potential biomarker candidates of some neuropsychiatric disorders.

Several animal studies document that stress can modify miRNA expression in different brain regions (Table 1). Both acute and chronic stress can influence the expression of various miRNAs and such molecular changes are likely to be similar in humans, because most miRNA families are phylogenetically conserved from C. elegans to humans [230,231].

Meerson et al. and Rinaldi et al. were the first to investigate region-specific alteration of miRNA upon stress exposure but they found contrasting data. In one study, chronic restraint stress caused significant changes of miRNA levels in two stress-responsive regions of the rat brain, the hippocampal CA1 and the central nucleus of the amygdala and most of these alterations were reductions (e.g., miR-134) [232]. In contrast, Rinaldi et al. observed an increase in the expression levels of miRNAs in the PFC following acute and repeated stress [233]. A more recent study used CMS exposure to investigate the miRNA expression profile in the hippocampus with a miRNA array chip technology and qRT-PCR analysis. Zhou et al. found that five miRNAs were significantly upregulated—miR-382-3p, miR-183-5p, miR-3573-5p, miR-202-3p, miR-493-3p, and only miR-370-3p was downregulated [234]. The gene ontology (GO) analysis of these miRNAs showed that their target genes may regulate many pathways that are involved in the development of MDD [234].

Alterations of miRNA expression in the PFC are likely to play a notable role in the pathogenesis of depression. A recent study reported an increased level of miR-9-5p, miR-128-1-5p, miR-382-5p and a decreased level of miR-16-5p, miR-129-5p, miR-219a-5p in the PFC of mice exposed to CMS [235]. The stress-induced increase of miR-9-5p and miR-128-1-5p was comparable to what has been reported in depressed patients [236,237].

CMS exposure can increase expression levels of miR-18a-5p, miR-34a-5p, miR-135a-5p, miR-195-5p, miR-320-3p, miR-674-3p, miR-872-5p in the ventral tegmental area (VTA) followed by parallel decrease of these miRNAs in the PFC of stress exposed rats and these alterations seem to be more pronounced in stress-resilient than anhedonic animals [238]. These miRNAs share a common target, namely the serotonin transporter (SERT), which is the pharmacological target of selective serotonin reuptake inhibitor (SSRI) antidepressants.

Zurawek et al. investigated miRNAs in stress resilient animals and found that 376 mature miRNAs measured in the periphery were associated with the resilient phenotype [239]. Furthermore, they reported that the serum concentration of miR-16 was associated with the stress-resilient phenotype as resilient animals had higher serum concentration of miR-16 compared to unstressed and anhedonic-like animals [239]. However, they did not find a correlation between miR-16 levels in the blood and in the brain in response to CMS [239]. They also observed that acute stress exposure resulted in the up-regulation of miR-16 in the ventral tegmental area of the stress-resilient animals, while miR-16 was down-regulated in the medial PFC of the stress-exposed animals [239]. One should emphasize here that miR-16 has an important role in the regulatory function of SERT, therefore miR-16 significantly contributes to the therapeutic action of the SSRIs [240]. 

Higuchi et al. were the first to demonstrate a crucial role of hippocampal miRNAs in the development of chronic stress-induced depression-like behavior and neuroplasticity. They observed a reduced level of hippocampal miR-124 in CMS treated mice [241]. Notably, the same study reported that neither viral-mediated hippocampal overexpression of miR-124, nor intrahippocampal infusion of a miR-124 inhibitor, affected depression-like behaviors in non-stressed mice [241]. In contrast, another study found that 21-days social defeat stress caused increased miR-124 levels in the rat hippocampus [243].

miR-134, a brain-specific miRNA, has a negative effect on synaptic development and plasticity [244]. A recent study found that chronic stress decreased the expression of miR-134 in the basolateral amygdala and this was associated with synaptic changes as well as decreased phosphorylation of CREB and decreased expression of BDNF [242].

In summary, there are increasing data available on the functional role of CNS-associated miRNAs in depressive disorders. So far, it is known that dysregulation of miRNAs can lead to silencing important target genes or even impaired mRNA translation causing diverse neuropathological outcomes. Clearly, more studies are needed to understand the role of specific miRNAs in the diagnosis and treatment of MDD.

## 10. Conclusions and Future Directions

The currently available experimental data provide convincing evidence that the CMS model has high translational value. However, more studies are needed to determine exactly which structural and molecular markers are useful in clinical practice for the diagnosis, prognostics and assessment of the therapeutic response. Future longitudinal studies using the CMS paradigm in combination with pharmacological treatment with clinically effective antidepressant drugs will allow investigations of essential cellular and molecular changes during disease progression and recovery. Such data should provide valuable and translational information on the pathophysiology of MDD.

## Figures and Tables

**Table 1:** Stress-induced changes in micro RNA levels.

Reference	miRNA		Analysis Method	Species	Brain Area	Behavioral Parameter
	Upregulated	Downregulated				
Meerson et al., 2010 [232]		miR-134	Spotted array, qRT-PCR	Adult rat	Hippocampal CA1, central nucleus of the amygdala	Chronic restraint stress
Rinaldi et al., 2010 [233]	let-7a, miR-9, miR-26a/b		Microarray, Northern blot	CD1 mice	PFC	Acute and chronic restraint stress
Zurawek et al., 2016 [239]	miR-16		qRT-PCR	Male Wistar Han rat	VTA	Chronic mild stress
		miR-16			medial PFC	
Higuchi et al., 2016 [241]		miR-124	qRT-PCR, Northern blot	BALB/c mice	Hippocampus	Chronic ultra-mild stress
Buran et al., 2017 [235]	miR-9-5p, miR-128-1-5p, miR-382-5p	miR-16-5p, miR-129-5p, miR-219a-5p	qRT-PCR	BALB/c mice	PFC	Chronic mild stress
Zurawek et al., 2017 [238]	miR-18a-5p, miR-34a-5p, miR-135a-5p, miR-195-5p, miR-320-3p, miR-674-3p, miR-872-5p		qRT-PCR	Wistar Han rat	VTA	Chronic mild stress
		miR-18a-5p, miR-34a-5p, miR-135a-5p, miR-195-5p, miR-320-3p, miR-674-3p, miR-872-5p			PFC	
Yu et al., 2018 [242]		miR-134	qRT-PCR	Wistar rat	BLA	Chronic unpredictable mild stress
Zhou et al., 2018 [234]	miR-382-3p, miR-183-5p, miR-3573-5p, miR-202-3p, miR-493-3p	miR-370-3p	Microarray, qRT-PCR	SPF Sprague-Dawley rat	Hippocampus	Chronic unpredictable mild stress

## References

[1] World Health Assembly (2012). 65 Global Burden of Mental Disorders and the Need for a Comprehensive, Coordinated Response from Health and Social Sectors at the Country Level: Report by the Secretariat.

[2] American Psychiatric Association (2013). Diagnostic and Statistical Manual of Mental Disorders.

[3] First M.B. (2013). DSM-5^®^ Handbook of Differential Diagnosis.

[4] Kempton M.J. (2011). Structural Neuroimaging Studies in Major Depressive Disorder. Arch. Gen. Psychiatry.

[5] Videbech P., Ravnkilde B. (2004). Reviews and Overviews Hippocampal Volume and Depression: A Meta-Analysis of MRI Studies. Hippocampal Vol. Depress. A Meta Anal. MRI Stud..

[6] Koolschijn P.C.M.P., Van Haren N.E.M., Lensvelt-Mulders G.J.L.M., Hulshoff Pol H.E., Kahn R.S. (2009). Brain volume abnormalities in major depressive disorder: A meta-analysis of magnetic resonance imaging studies. Hum. Brain Mapp..

[7] Bromis K., Calem M., Reinders A.A.T.S., Williams S.C.R., Kempton M.J. (2018). Meta-Analysis of 89 Structural MRI studies in posttraumatic stress disorder and comparison with major depressive disorder. Am. J. Psychiatry.

[8] Roddy D.W., Farrell C., Doolin K., Roman E., Tozzi L., Frodl T., O’Keane V., O’Hanlon E. (2019). The Hippocampus in Depression: More Than the Sum of Its Parts? Advanced Hippocampal Substructure Segmentation in Depression. Biol. Psychiatry.

[9] Czéh B., Lucassen P.J. (2007). What causes the hippocampal volume decrease in depression? Are neurogenesis, glial changes and apoptosis implicated?. Eur. Arch. Psychiatry Clin. Neurosci..

[10] MacQueen G., Frodl T. (2011). The hippocampus in major depression: Evidence for the convergence of the bench and bedside in psychiatric research. Mol. Psychiatry.

[11] Malykhin N.V., Coupland N.J. (2015). Hippocampal neuroplasticity in major depressive disorder. Neuroscience.

[12] Pittenger C., Duman R.S. (2008). Stress, depression, and neuroplasticity: A convergence of mechanisms. Neuropsychopharmacology.

[13] Czéh B., Fuchs E., Wiborg O., Simon M. (2016). Animal models of major depression and their clinical implications. Prog. Neuro Psychopharmacol. Biol. Psychiatry.

[14] Willner P., Towell A., Sampson D., Sophokleous S., Muscat R. (1987). Reduction of sucrose preference by chronic unpredictable mild stress, and its restoration by a tricyclic antidepressant. Psychopharmacology (Berlin).

[15] Willner P. (1997). Validity, reliability and utility of the chronic mild stress model of depression: A 10-year review and evaluation. Psychopharmacology (Berlin).

[16] Willner P. (2017). The chronic mild stress (CMS) model of depression: History, evaluation and usage. Neurobiol. Stress.

[17] Wiborg O. (2013). Chronic mild stress for modeling anhedonia. Cell Tissue Res..

[18] Antoniuk S., Bijata M., Ponimaskin E., Wlodarczyk J. (2019). Chronic unpredictable mild stress for modeling depression in rodents: Meta-analysis of model reliability. Neurosci. Biobehav. Rev..

[19] Vollmayr B., Gass P. (2013). Learned helplessness: Unique features and translational value of a cognitive depression model. Cell Tissue Res..

[20] Koo J.W., Chaudhury D., Han M.H., Nestler E.J. (2019). Role of Mesolimbic Brain-Derived Neurotrophic Factor in Depression. Biol. Psychiatry.

[21] Bergström A., Jayatissa M.N., Mørk A., Wiborg O. (2008). Stress sensitivity and resilience in the chronic mild stress rat model of depression; an in situ hybridization study. Brain Res..

[22] Martis L.S., Brision C., Holmes M.C., Wiborg O. (2018). Resilient and depressive-like rats show distinct cognitive impairments in the touchscreen paired-associates learning (PAL) task. Neurobiol. Learn. Mem..

[23] Czéh B., Vardya I., Varga Z., Febbraro F., Csabai D., Martis L.S., Højgaard K., Henningsen K., Bouzinova E.V., Miseta A. (2018). Long-term stress disrupts the structural and functional integrity of GABAergic neuronal networks in the medial prefrontal cortex of rats. Front. Cell. Neurosci..

[24] Jayatissa M.N., Bisgaard C., Tingström A., Papp M., Wiborg O. (2006). Hippocampal cytogenesis correlates to escitalopram-mediated recovery in a chronic mild stress rat model of depression. Neuropsychopharmacology.

[25] Belleau E.L., Treadway M.T., Pizzagalli D.A. (2019). The Impact of Stress and Major Depressive Disorder on Hippocampal and Medial Prefrontal Cortex Morphology. Biol. Psychiatry.

[26] Nestler E.J., Barrot M., DiLeone R.J., Eisch A.J., Gold S.J., Monteggia L.M. (2002). Neurobiology of depression. Neuron.

[27] Krishnan V., Nestler E.J. (2008). The molecular neurobiology of depression. Nature.

[28] Khan A.R., Kroenke C.D., Wiborg O., Chuhutin A., Nyengaard J.R., Hansen B., Jespersen S.N. (2018). Differential microstructural alterations in rat cerebral cortex in a model of chronic mild stress depression. PLoS ONE.

[29] Khan A.R., Hansen B., Danladi J., Chuhutin A., Wiborg O., Nyengaard J.R., Jespersen S.N. (2019). Neurite atrophy in dorsal hippocampus of rat indicates incomplete recovery of chronic mild stress induced depression. NMR Biomed..

[30] Khan A.R., Hansen B., Wiborg O., Kroenke C.D., Jespersen S.N. (2018). Diffusion MRI and MR spectroscopy reveal microstructural and metabolic brain alterations in chronic mild stress exposed rats: A CMS recovery study. Neuroimage.

[31] Herman J.P., Ostrander M.M., Mueller N.K., Figueiredo H. (2005). Limbic system mechanisms of stress regulation: Hypothalamo-pituitary- adrenocortical axis. Prog. Neuro Psychopharmacol. Biol. Psychiatry.

[32] McEwen B.S., De Kloet E.R., Rostene W. (1986). Adrenal steroid receptors and actions in the nervous system. Physiol. Rev..

[33] McEwen B.S. (1999). Stress and hippocampal plasticity. Annu. Rev. Neurosci..

[34] McEwen B.S., Akil H. (2020). Revisiting the stress concept: Implications for affective disorders. J. Neurosci..

[35] Cohen H., Kozlovsky N., Matar M.A., Zohar J., Kaplan Z. (2014). Distinctive hippocampal and amygdalar cytoarchitectural changes underlie specific patterns of behavioral disruption following stress exposure in an animal model of PTSD. Eur. Neuropsychopharmacol..

[36] Lucassen P.J., Pruessner J., Sousa N., Almeida O.F.X., Van Dam A.M., Rajkowska G., Swaab D.F., Czéh B. (2014). Neuropathology of stress. Acta Neuropathol..

[37] Lucassen P.J., Vollmann-Honsdorf G.K., Gleisberg M., Czéh B., De Kloet E.R., Fuchs E. (2001). Chronic psychosocial stress differentially affects apoptosis in hippocampal subregions and cortex of the adult tree shrew. Eur. J. Neurosci..

[38] Lucassen P.J., Heine V., Muller M., van der Beek E., Wiegant V., De Kloet R.E., Joels M., Fuchs E., Swaab D., Czeh B. (2006). Stress, Depression and Hippocampal Apoptosis. CNS Neurol. Disord. Drug Targets.

[39] Khan A.R., Chuhutin A., Wiborg O., Kroenke C.D., Nyengaard J.R., Hansen B., Jespersen S.N. (2016). Biophysical modeling of high field diffusion MRI demonstrates micro-structural aberration in chronic mild stress rat brain. Neuroimage.

[40] Khan A.R., Chuhutin A., Wiborg O., Kroenke C.D., Nyengaard J.R., Hansen B., Jespersen S.N. (2016). Summary of high field diffusion MRI and microscopy data demonstrate microstructural aberration in chronic mild stress rat brain. Data Br..

[41] Khan A.R., Jespersen S.N., Wiborg O., Kroenke C., Hansen B. (2018). Microstructural and metabolic recovery of anhedonic rat brains: An in vivo diffusion MRI and 1H-MRS approach. Data.

[42] Sousa N., Lukoyanov N.V., Madeira M.D., Almeida O.F.X., Paula-Barbosa M.M. (2000). Reorganization of the morphology of hippocampal neurites and synapses after stress-induced damage correlates with behavioral improvement. Neuroscience.

[43] Morais M., Santos P.A.R., Mateus-Pinheiro A., Patrício P., Pinto L., Sousa N., Pedroso P., Almeida S., Filipe A., Bessa J.M. (2014). The effects of chronic stress on hippocampal adult neurogenesis and dendritic plasticity are reversed by selective MAO-A inhibition. J. Psychopharmacol..

[44] Qiao H., An S.C., Xu C., Ma X.M. (2017). Role of proBDNF and BDNF in dendritic spine plasticity and depressive-like behaviors induced by an animal model of depression. Brain Res..

[45] Pinto V., Costa J.C., Morgado P., Mota C., Miranda A., Bravo F.V., Oliveira T.G., Cerqueira J.J., Sousa N. (2015). Differential impact of chronic stress along the hippocampal dorsal-ventral axis. Brain Struct. Funct..

[46] Zhuang P.C., Tan Z.N., Jia Z.Y., Wang B., Grady J.J., Ma X.M. (2019). Treadmill exercise reverses depression model-induced alteration of dendritic spines in the brain areas of mood circuit. Front. Behav. Neurosci..

[47] Jayatissa M.N., Henningsen K., West M.J., Wiborg O. (2009). Decreased cell proliferation in the dentate gyrus does not associate with development of anhedonic-like symptoms in rats. Brain Res..

[48] Lee K.J., Kim S.J., Kim S.W., Choi S.H., Shin Y.C., Park S.H., Moon B.H., Cho E., Lee M.S., Choi S.H. (2006). Chronic mild stress decreases survival, but not proliferation, of new-born cells in adult rat hippocampus. Exp. Mol. Med..

[49] Mineur Y.S., Belzung C., Crusio W.E. (2007). Functional implications of decreases in neurogenesis following chronic mild stress in mice. Neuroscience.

[50] Zhou Q.G., Hu Y., Hua Y., Hu M., Luo C.X., Han X., Zhu X.J., Wang B., Xu J.S., Zhu D.Y. (2007). Neuronal nitric oxide synthase contributes to chronic stress-induced depression by suppressing hippocampal neurogenesis. J. Neurochem..

[51] Goshen I., Kreisel T., Ben-Menachem-Zidon O., Licht T., Weidenfeld J., Ben-Hur T., Yirmiya R. (2008). Brain interleukin-1 mediates chronic stress-induced depression in mice via adrenocortical activation and hippocampal neurogenesis suppression. Mol. Psychiatry.

[52] Toth E., Gersner R., Wilf-Yarkoni A., Raizel H., Dar D.E., Richter-Levin G., Levit O., Zangen A. (2008). Age-dependent effects of chronic stress on brain plasticity and depressive behavior. J. Neurochem..

[53] Tanti A., Rainer Q., Minier F., Surget A., Belzung C. (2012). Differential environmental regulation of neurogenesis along the septo-temporal axis of the hippocampus. Neuropharmacology.

[54] Tanti A., Westphal W.P., Girault V., Brizard B., Devers S., Leguisquet A.M., Surget A., Belzung C. (2013). Region-dependent and stage-specific effects of stress, environmental enrichment, and antidepressant treatment on hippocampal neurogenesis. Hippocampus.

[55] De Andrade J.S., Céspedes I.C., Abrão R.O., dos Santos T.B., Diniz L., Britto L.R.G., Spadari-Bratfisch R.C., Ortolani D., Melo-Thomas L., da Silva R.C.B. (2013). Chronic unpredictable mild stress alters an anxiety-related defensive response, Fos immunoreactivity and hippocampal adult neurogenesis. Behav. Brain Res..

[56] Parihar V.K., Hattiangady B., Shuai B., Shetty A.K. (2013). Mood and memory deficits in a model of gulf war illness are linked with reduced neurogenesis, partial neuron loss, and mild inflammation in the hippocampus. Neuropsychopharmacology.

[57] Alves N.D., Correia J.S., Patrício P., Mateus-Pinheiro A., Machado-Santos A.R., Loureiro-Campos E., Morais M., Bessa J.M., Sousa N., Pinto L. (2017). Adult hippocampal neuroplasticity triggers susceptibility to recurrent depression. Transl. Psychiatry.

[58] Culig L., Surget A., Bourdey M., Khemissi W., Le Guisquet A.M., Vogel E., Sahay A., Hen R., Belzung C. (2017). Increasing adult hippocampal neurogenesis in mice after exposure to unpredictable chronic mild stress may counteract some of the effects of stress. Neuropharmacology.

[59] Tunc-Ozcan E., Peng C.Y., Zhu Y., Dunlop S.R., Contractor A., Kessler J.A. (2019). Activating newborn neurons suppresses depression and anxiety-like behaviors. Nat. Commun..

[60] Jayatissa M.N., Bisgaard C.F., West M.J., Wiborg O. (2008). The number of granule cells in rat hippocampus is reduced after chronic mild stress and re-established after chronic escitalopram treatment. Neuropharmacology.

[61] Jayatissa M.N., Henningsen K., Nikolajsen G., West M.J., Wiborg O. (2010). A reduced number of hippocampal granule cells does not associate with an anhedonia-like phenotype in a rat chronic mild stress model of depression. Stress.

[62] Yang C., Wang G., Wang H., Liu Z., Wang X. (2009). Cytoskeletal alterations in rat hippocampus following chronic unpredictable mild stress and re-exposure to acute and chronic unpredictable mild stress. Behav. Brain Res..

[63] De Kloet E.R., Joëls M., Holsboer F. (2005). Stress and the brain: From adaptation to disease. Nat. Rev. Neurosci..

[64] Oitzl M.S., Champagne D.L., van der Veen R., de Kloet E.R. (2010). Brain development under stress: Hypotheses of glucocorticoid actions revisited. Neurosci. Biobehav. Rev..

[65] Sapolsky R.M., Krey L.C., McEwen B.S. (1986). The neuroendocrinology of stress and aging: The glucocorticoid cascade hypothesis. Endocr. Rev..

[66] Czeh B., Simon M., Van Der Hart M.G.C., Schmelting B., Hesselink M.B., Fuchs E. (2005). Chronic stress decreases the number of parvalbumin-immunoreactive interneurons in the hippocampus: Prevention by treatment with a substance P receptor (NK1) antagonist. Neuropsychopharmacology.

[67] Hu H., Su L., Xu Y.Q., Zhang H., Wang L.W. (2010). Behavioral and [F-18] fluorodeoxyglucose micro positron emission tomography imaging study in a rat chronic mild stress model of depression. Neuroscience.

[68] Gilabert-Juan J., Bueno-Fernandez C., Castillo-Gomez E., Nacher J. (2017). Reduced interneuronal dendritic arborization in CA1 but not in CA3 region of mice subjected to chronic mild stress. Brain Behav..

[69] Banasr M., Lepack A., Fee C., Duric V., Maldonado-Aviles J., DiLeone R., Sibille E., Duman R.S., Sanacora G. (2017). Characterization of GABAergic Marker Expression in the Chronic Unpredictable Stress Model of Depression. Chron. Stress.

[70] Czéh B., Varga Z.K.K., Henningsen K., Kovács G.L., Miseta A., Wiborg O. (2015). Chronic stress reduces the number of GABAergic interneurons in the adult rat hippocampus, dorsal-ventral and region-specific differences. Hippocampus.

[71] Czéh B., Ábrahám H., Tahtakran S., Houser C.R., Seress L. (2013). Number and regional distribution of GAD65 mRNA-expressing interneurons in the rat hippocampal formation. Acta Biol. Hung..

[72] Roux L., Buzsáki G. (2015). Tasks for inhibitory interneurons in intact brain circuits. Neuropharmacology.

[73] Pelkey K.A., Chittajallu R., Craig M.T., Tricoire L., Wester J.C., McBain C.J. (2017). Hippocampal gabaergic inhibitory interneurons. Physiol. Rev..

[74] Ren Z., Pribiag H., Jefferson S.J., Shorey M., Fuchs T., Stellwagen D., Luscher B. (2016). Bidirectional Homeostatic Regulation of a Depression-Related Brain State by Gamma-Aminobutyric Acidergic Deficits and Ketamine Treatment. Biol. Psychiatry.

[75] Duman R.S., Sanacora G., Krystal J.H. (2019). Altered Connectivity in Depression: GABA and Glutamate Neurotransmitter Deficits and Reversal by Novel Treatments. Neuron.

[76] Fogaça M.V., Duman R.S. (2019). Cortical GABAergic dysfunction in stress and depression: New insights for therapeutic interventions. Front. Cell. Neurosci..

[77] Hu W., Zhang M., Czéh B., Flügge G., Zhang W. (2010). Stress impairs GABAergic network function in the hippocampus by activating nongenomic glucocorticoid receptors and affecting the integrity of the parvalbumin-expressing neuronal network. Neuropsychopharmacology.

[78] Rossetti A.C., Paladini M.S., Colombo M., Gruca P., Lason-Tyburkiewicz M., Tota-Glowczyk K., Papp M., Riva M.A., Molteni R. (2018). Chronic Stress Exposure Reduces Parvalbumin Expression in the Rat Hippocampus through an Imbalance of Redox Mechanisms: Restorative Effect of the Antipsychotic Lurasidone. Int. J. Neuropsychopharmacol..

[79] Csabai D., Seress L., Varga Z., Ábrahám H., Miseta A., Wiborg O., Czéh B. (2017). Electron Microscopic Analysis of Hippocampal Axo-Somatic Synapses in a Chronic Stress Model for Depression. Hippocampus.

[80] Magarinos A.M., Verdugo J.M.G., McEwen B.S. (1997). Chronic stress alters synaptic terminal structure in hippocampus. Proc. Natl. Acad. Sci. USA.

[81] Stewart M.G., Davies H.A., Sandi C., Kraev I.V., Rogachevsky V.V., Peddie C.J., Rodriguez J.J., Cordero M.I., Donohue H.S., Gabbott P.L.A. (2005). Stress suppresses and learning induces plasticity in CA3 of rat hippocampus: A three-dimensional ultrastructural study of thorny excrescences and their postsynaptic densities. Neuroscience.

[82] Donohue H.S., Gabbott P.L.A., Davies H.A., Rodríguez J.J., Cordero M.I., Sandi C., Medvedev N.I., Popov V.I., Colyer F.M., Peddie C.J. (2006). Chronic restraint stress induces changes in synapse morphology in stratum lacunosum-moleculare CA1 rat hippocampus: A stereological and three-dimensional ultrastructural study. Neuroscience.

[83] Hajszan T., Dow A., Warner-Schmidt J.L., Szigeti-Buck K., Sallam N.L., Parducz A., Leranth C., Duman R.S. (2009). Remodeling of Hippocampal Spine Synapses in the Rat Learned Helplessness Model of Depression. Biol. Psychiatry.

[84] Li X.-L., Yuan Y.-G., Xu H., Wu D., Gong W.-G., Geng L.-Y., Wu F.-F., Tang H., Xu L., Zhang Z.-J. (2015). Changed Synaptic Plasticity in Neural Circuits of Depressive-Like and Escitalopram-Treated Rats. Int. J. Neuropsychopharmacol..

[85] Hei M., Chen P., Wang S., Li X., Xu M., Zhu X., Wang Y., Duan J., Huang Y., Zhao S. (2019). Effects of chronic mild stress induced depression on synaptic plasticity in mouse hippocampus. Behav. Brain Res..

[86] Holm M.M., Nieto-Gonzalez J.L., Vardya I., Henningsen K., Jayatissa M.N., Wiborg O., Jensen K. (2011). Hippocampal GABAergic dysfunction in a rat chronic mild stress model of depression. Hippocampus.

[87] Hu W., Zhang M., Czéh B., Zhang W., Flügge G. (2011). Chronic restraint stress impairs endocannabinoid mediated suppression of GABAergic signaling in the hippocampus of adult male rats. Brain Res. Bull..

[88] Reich C.G., Mihalik G.R., Iskander A.N., Seckler J.C., Weiss M.S. (2013). Adolescent chronic mild stress alters hippocampal CB1 receptor-mediated excitatory neurotransmission and plasticity. Neuroscience.

[89] Qiao H., An S.C., Ren W., Ma X.M. (2014). Progressive alterations of hippocampal CA3-CA1 synapses in an animal model of depression. Behav. Brain Res..

[90] Bannerman D.M., Sprengel R., Sanderson D.J., Mchugh S.B., Rawlins J.N.P., Monyer H., Seeburg P.H. (2014). Hippocampal synaptic plasticity, spatial memory and anxiety. Nat. Rev. Neurosci..

[91] Fanselow M.S., Dong H.W. (2010). Are the Dorsal and Ventral Hippocampus Functionally Distinct Structures?. Neuron.

[92] Anacker C., Hen R. (2017). Adult hippocampal neurogenesis and cognitive flexibility-linking memory and mood. Nat. Rev. Neurosci..

[93] Delgado Y., Palacios R., Campo A., Henningsen K., Verhoye M., Poot D., Dijkstra J., Van Audekerke J., Benveniste H., Sijbers J. (2011). Magnetic resonance imaging and spectroscopy reveal differential hippocampal changes in anhedonic and resilient subtypes of the chronic mild stress rat model. Biol. Psychiatry.

[94] Stein-Behrens B.A., Lin W.J., Sapolsky R.M. (1994). Physiological Elevations of Glucocorticoids Potentiate Glutamate Accumulation in the Hippocampus. J. Neurochem..

[95] Magalhães R., Novais A., Barrie‘re D.A., Marques P., Marques F., Sousa J.C., Cerqueira J.J., Cachia A., Jay T.M., Bottlaender M. (2019). A resting-state functional MR imaging and spectroscopy study of the dorsal hippocampus in the chronic unpredictable stress rat model. J. Neurosci..

[96] Sapolsky R.M. (2000). The possibility of neurotoxicity in the hippocampus in major depression: A primer on neuron death. Biol. Psychiatry.

[97] Dantzer R., Walker A.K. (2014). Is there a role for glutamate-mediated excitotoxicity in inflammation-induced depression?. J. Neural Transm..

[98] Czéh B., Simon M., Schmelting B., Hiemke C., Fuchs E. (2006). Astroglial plasticity in the hippocampus is affected by chronic psychosocial stress and concomitant fluoxetine treatment. Neuropsychopharmacology.

[99] Miyata S., Taniguchi M., Koyama Y., Shimizu S., Tanaka T., Yasuno F., Yamamoto A., Iida H., Kudo T., Katayama T. (2016). Association between chronic stress-induced structural abnormalities in Ranvier nodes and reduced oligodendrocyte activity in major depression. Sci. Rep..

[100] Tynan R.J., Naicker S., Hinwood M., Nalivaiko E., Buller K.M., Pow D.V., Day T.A., Walker F.R. (2010). Chronic stress alters the density and morphology of microglia in a subset of stress-responsive brain regions. Brain Behav. Immun..

[101] Czéh B., Nagy S.A. (2018). Clinical findings documenting cellular and molecular abnormalities of glia in depressive disorders. Front. Mol. Neurosci..

[102] Li L.F., Yang J., Ma S.P., Qu R. (2013). Magnolol treatment reversed the glial pathology in an unpredictable chronic mild stress-induced rat model of depression. Eur. J. Pharmacol..

[103] Machado-Santos A.R., Alves N.D., Araújo B., Correia J.S., Patrício P., Mateus-Pinheiro A., Loureiro-Campos E., Bessa J.M., Sousa N., Pinto L. (2019). Astrocytic plasticity at the dorsal dentate gyrus on an animal model of recurrent depression. Neuroscience.

[104] Wang Y.L., Han Q.Q., Gong W.Q., Pan D.H., Wang L.Z., Hu W., Yang M., Li B., Yu J., Liu Q. (2018). Microglial activation mediates chronic mild stress-induced depressive- and anxiety-like behavior in adult rats. J. Neuroinflammation.

[105] Ferle V., Repouskou A., Aspiotis G., Raftogianni A., Chrousos G., Stylianopoulou F., Stamatakis A. (2020). Synergistic effects of early life mild adversity and chronic social defeat on rat brain microglia and cytokines. Physiol. Behav..

[106] Boda E. (2019). Myelin and oligodendrocyte lineage cell dysfunctions: New players in the etiology and treatment of depression and stress-related disorders. Eur. J. Neurosci..

[107] Cobb J.A., O’Neill K., Milner J., Mahajan G.J., Lawrence T.J., May W.L., Miguel-Hidalgo J., Rajkowska G., Stockmeier C.A. (2016). Density of GFAP-immunoreactive astrocytes is decreased in left hippocampi in major depressive disorder. Neuroscience.

[108] Innes S., Pariante C.M., Borsini A. (2019). Microglial-driven changes in synaptic plasticity: A possible role in major depressive disorder. Psychoneuroendocrinology.

[109] Wang Q., Jie W., Liu J.-H., Yang J.-M., Gao T.-M. (2017). An astroglial basis of major depressive disorder? An overview. Glia.

[110] Murakami S., Imbe H., Morikawa Y., Kubo C., Senba E. (2005). Chronic stress, as well as acute stress, reduces BDNF mRNA expression in the rat hippocampus but less robustly. Neurosci. Res..

[111] Tornese P., Sala N., Bonini D., Bonifacino T., La Via L., Milanese M., Treccani G., Seguini M., Ieraci A., Mingardi J. (2019). Chronic mild stress induces anhedonic behavior and changes in glutamate release, BDNF trafficking and dendrite morphology only in stress vulnerable rats. The rapid restorative action of ketamine. Neurobiol. Stress.

[112] Felger J.C., Lotrich F.E. (2013). Inflammatory cytokines in depression: Neurobiological mechanisms and therapeutic implications. Neuroscience.

[113] Chattarji S., Tomar A., Suvrathan A., Ghosh S., Rahman M.M. (2015). Neighborhood matters: Divergent patterns of stress-induced plasticity across the brain. Nat. Neurosci..

[114] Christensen T., Bisgaard C.F., Wiborg O. (2011). Biomarkers of anhedonic-like behavior, antidepressant drug refraction, and stress resilience in a rat model of depression. Neuroscience.

[115] Duman R.S., Monteggia L.M. (2006). A Neurotrophic Model for Stress-Related Mood Disorders. Biol. Psychiatry.

[116] Groves J.O. (2007). Is it time to reassess the BDNF hypothesis of depression?. Mol. Psychiatry.

[117] Martinowich K., Manji H., Lu B. (2007). New insights into BDNF function in depression and anxiety. Nat. Neurosci..

[118] Soliman M.A., Aboharb F., Zeltner N., Studer L. (2017). Pluripotent stem cells in neuropsychiatric disorders. Mol. Psychiatry.

[119] Bardy C., Greenberg Z., Perry S.W., Licinio J. (2020). Personalized psychiatry with human iPSCs and neuronal reprogramming. Personalized Psychiatry.

[120] Meijer M., Rehbach K., Brunner J.W., Classen J.A., Lammertse H.C.A., van Linge L.A., Schut D., Krutenko T., Hebisch M., Cornelisse L.N. (2019). A Single-Cell Model for Synaptic Transmission and Plasticity in Human iPSC-Derived Neurons. Cell Rep..

[121] Arnsten A.F.T. (2009). Stress signalling pathways that impair prefrontal cortex structure and function. Nat. Rev. Neurosci..

[122] Herman J.P., Prewitt C.M.-F., Cullinan W.E. (1996). Neuronal Circuit Regulation of the Hypothalamo-Pituitary-Adrenocortical Stress Axis. Crit. Rev. Neurobiol..

[123] Drevets W.C., Price J.L., Simpson J.R., Todd R.D., Reich T., Vannier M., Raichle M.E. (1997). Subgenual prefrontal cortex abnormalities in mood disorders. Nature.

[124] Drevets W.C. (2000). Neuroimaging studies of mood disorders. Biol. Psychiatry.

[125] Drevets W.C. (2000). Functional anatomical abnormalities in limbic and prefrontal cortical structures in major depression. Prog. Brain Res..

[126] McEwen B.S., Morrison J.H. (2013). The Brain on Stress: Vulnerability and Plasticity of the Prefrontal Cortex over the Life Course. Neuron.

[127] Ansell E.B., Rando K., Tuit K., Guarnaccia J., Sinha R. (2012). Cumulative adversity and smaller gray matter volume in medial prefrontal, anterior cingulate, and insula regions. Biol. Psychiatry.

[128] Moreno G.L., Bruss J., Denburg N.L. (2017). Increased perceived stress is related to decreased prefrontal cortex volumes among older adults. J. Clin. Exp. Neuropsychol..

[129] Savic I., Perski A., Osika W. (2018). MRI Shows that Exhaustion Syndrome Due to Chronic Occupational Stress is Associated with Partially Reversible Cerebral Changes. Cereb. Cortex.

[130] Csabai D., Wiborg O., Czéh B. (2018). Reduced synapse and axon numbers in the prefrontal cortex of rats subjected to a chronic stress model for depression. Front. Cell. Neurosci..

[131] Banasr M., Valentine G.W., Li X.Y., Gourley S.L., Taylor J.R., Duman R.S. (2007). Chronic Unpredictable Stress Decreases Cell Proliferation in the Cerebral Cortex of the Adult Rat. Biol. Psychiatry.

[132] Czéh B., Perez-Cruz C., Fuchs E., Flügge G. (2008). Chronic stress-induced cellular changes in the medial prefrontal cortex and their potential clinical implications: Does hemisphere location matter?. Behav. Brain Res..

[133] Bachis A., Cruz M.I., Nosheny R.L., Mocchetti I. (2008). Chronic unpredictable stress promotes neuronal apoptosis in the cerebral cortex. Neurosci. Lett..

[134] Dias-Ferreira E., Sousa J.C., Melo I., Morgado P., Mesquita A.R., Cerqueira J.J., Costa R.M., Sousa N. (2009). Chronic stress causes frontostriatal reorganization and affects decision-making. Science (80-. ).

[135] Kafetzopoulos V., Kokras N., Sotiropoulos I., Oliveira J.F., Leite-Almeida H., Vasalou A., Sardinha V.M., Papadopoulou-Daifoti Z., Almeida O.F.X., Antoniou K. (2017). The nucleus reuniens: A key node in the neurocircuitry of stress and depression. Mol. Psychiatry.

[136] Li N., Liu R.J., Dwyer J.M., Banasr M., Lee B., Son H., Li X.Y., Aghajanian G., Duman R.S. (2011). Glutamate N-methyl-D-aspartate receptor antagonists rapidly reverse behavioral and synaptic deficits caused by chronic stress exposure. Biol. Psychiatry.

[137] Cerqueira J.J., Taipa R., Uylings H.B.M., Almeida O.F.X., Sousa N. (2007). Specific configuration of dendritic degeneration in pyramidal neurons of the medial prefrontal cortex induced by differing corticosteroid regimens. Cereb. Cortex.

[138] Hemanth Kumar B.S., Mishra S.K., Rana P., Singh S., Khushu S. (2012). Neurodegenerative evidences during early onset of depression in CMS rats as detected by proton magnetic resonance spectroscopy at 7T. Behav. Brain Res..

[139] McKlveen J.M., Moloney R.D., Scheimann J.R., Myers B., Herman J.P. (2019). “Braking” the Prefrontal Cortex: The Role of Glucocorticoids and Interneurons in Stress Adaptation and Pathology. Biol. Psychiatry.

[140] Duman R.S., Aghajanian G.K., Sanacora G., Krystal J.H. (2016). Synaptic plasticity and depression: New insights from stress and rapid-acting antidepressants. Nat. Med..

[141] Ghosal S., Hare B.D., Duman R.S. (2017). Prefrontal cortex GABAergic deficits and circuit dysfunction in the pathophysiology and treatment of chronic stress and depression. Curr. Opin. Behav. Sci..

[142] McKlveen J.M., Morano R.L., Fitzgerald M., Zoubovsky S., Cassella S.N., Scheimann J.R., Ghosal S., Mahbod P., Packard B.A., Myers B. (2016). Chronic Stress Increases Prefrontal Inhibition: A Mechanism for Stress-Induced Prefrontal Dysfunction. Biol. Psychiatry.

[143] Gilabert-Juan J., Castillo-Gomez E., Guirado R., Moltó M.D., Nacher J. (2013). Chronic stress alters inhibitory networks in the medial prefrontal cortex of adult mice. Brain Struct. Funct..

[144] Shepard R., Page C.E., Coutellier L. (2016). Sensitivity of the prefrontal GABAergic system to chronic stress in male and female mice: Relevance for sex differences in stress-related disorders. Neuroscience.

[145] Shepard R., Coutellier L. (2018). Changes in the Prefrontal Glutamatergic and Parvalbumin Systems of Mice Exposed to Unpredictable Chronic Stress. Mol. Neurobiol..

[146] Page C.E., Shepard R., Heslin K., Coutellier L. (2019). Prefrontal parvalbumin cells are sensitive to stress and mediate anxiety-related behaviors in female mice. Sci. Rep..

[147] Maguire J. (2019). Neuroactive Steroids and GABAergic Involvement in the Neuroendocrine Dysfunction Associated With Major Depressive Disorder and Postpartum Depression. Front. Cell. Neurosci..

[148] Kang H.J., Voleti B., Hajszan T., Rajkowska G., Stockmeier C.A., Licznerski P., Lepack A., Majik M.S., Jeong L.S., Banasr M. (2012). Decreased expression of synapse-related genes and loss of synapses in major depressive disorder. Nat. Med..

[149] Holmes S.E., Scheinost D., Finnema S.J., Naganawa M., Davis M.T., DellaGioia N., Nabulsi N., Matuskey D., Angarita G.A., Pietrzak R.H. (2019). Lower synaptic density is associated with depression severity and network alterations. Nat. Commun..

[150] Banasr M., Duman R.S. (2008). Glial Loss in the Prefrontal Cortex Is Sufficient to Induce Depressive-like Behaviors. Biol. Psychiatry.

[151] Banasr M., Chowdhury G.M.I., Terwilliger R., Newton S.S., Duman R.S., Behar K.L., Sanacora G. (2010). Glial pathology in an animal model of depression: Reversal of stress-induced cellular, metabolic and behavioral deficits by the glutamate-modulating drug riluzole. Mol. Psychiatry.

[152] Hinwood M., Morandini J., Day T.A., Walker F.R. (2012). Evidence that Microglia Mediate the Neurobiological Effects of Chronic Psychological Stress on the Medial Prefrontal Cortex. Cereb. Cortex.

[153] Hinwood M., Tynan R.J., Charnley J.L., Beynon S.B., Day T.A., Rohan Walker F. (2013). Chronic Stress Induced Remodeling of the Prefrontal Cortex: Structural Re-Organization of Microglia and the Inhibitory Effect of Minocycline. Cereb. Cortex.

[154] Tynan R.J., Beynon S.B., Hinwood M., Johnson S.J., Nilsson M., Woods J.J., Walker F.R. (2013). Chronic stress-induced disruption of the astrocyte network is driven by structural atrophy and not loss of astrocytes. Acta Neuropathol..

[155] Bollinger J.L., Bergeon Burns C.M., Wellman C.L. (2016). Differential effects of stress on microglial cell activation in male and female medial prefrontal cortex. Brain. Behav. Immun..

[156] Lehmann M.L., Weigel T.K., Elkahloun A.G., Herkenham M. (2017). Chronic social defeat reduces myelination in the mouse medial prefrontal cortex. Sci. Rep..

[157] Wohleb E.S., Terwilliger R., Duman C.H., Duman R.S. (2018). Stress-Induced Neuronal Colony Stimulating Factor 1 Provokes Microglia-Mediated Neuronal Remodeling and Depressive-like Behavior. Biol. Psychiatry.

[158] Cathomas F., Azzinnari D., Bergamini G., Sigrist H., Buerge M., Hoop V., Wicki B., Goetze L., Soares S., Kukelova D. (2019). Oligodendrocyte gene expression is reduced by and influences effects of chronic social stress in mice. Genes Brain Behav..

[159] Rajkowska G., Miguel-Hidalgo J.J., Wei J., Dilley G., Pittman S.D., Meltzer H.Y., Overholser J.C., Roth B.L., Stockmeier C.A. (1999). Morphometric evidence for neuronal and glial prefrontal cell pathology in major depression∗∗See accompanying Editorial, in this issue. Biol. Psychiatry.

[160] Rajkowska G. (2000). Postmortem studies in mood disorders indicate altered numbers of neurons and glial cells. Biol. Psychiatry.

[161] Bender C.L., Calfa G.D., Molina V.A. (2016). Astrocyte plasticity induced by emotional stress: A new partner in psychiatric physiopathology?. Prog. Neuro Psychopharmacol. Biol. Psychiatry.

[162] Liu J., Dietz K., Hodes G.E., Russo S.J., Casaccia P. (2018). Widespread transcriptional alternations in oligodendrocytes in the adult mouse brain following chronic stress. Dev. Neurobiol..

[163] Canbeyli R. (2010). Sensorimotor modulation of mood and depression: An integrative review. Behav. Brain Res..

[164] Buyukdura J.S., McClintock S.M., Croarkin P.E. (2011). Psychomotor retardation in depression: Biological underpinnings, measurement, and treatment. Prog. Neuro Psychopharmacol. Biol. Psychiatry.

[165] Bracht T., Federspiel A., Schnell S., Horn H., Höfle O., Wiest R., Dierks T., Strik W., Müller T.J., Walther S. (2012). Cortico-Cortical White Matter Motor Pathway Microstructure Is Related to Psychomotor Retardation in Major Depressive Disorder. PLoS ONE.

[166] Yin Y., Wang M., Wang Z., Xie C., Zhang H., Zhang H., Zhang Z., Yuan Y. (2018). Decreased cerebral blood flow in the primary motor cortex in major depressive disorder with psychomotor retardation. Prog. Neuro Psychopharmacol. Biol. Psychiatry.

[167] Zhang H., Li L., Wu M., Chen Z., Hu X., Chen Y., Zhu H., Jia Z., Gong Q. (2016). Brain gray matter alterations in first episodes of depression: A meta-analysis of whole-brain studies. Neurosci. Biobehav. Rev..

[168] Kähkönen S., Yamashita H., Rytsälä H., Suominen K., Ahveninen J., Isometsä E. (2007). Dysfunction in early auditory processing in major depressive disorder revealed by combined MEG and EEG. J Psychiatry Neurosci..

[169] Smiley J.F., Hackett T.A., Bleiwas C., Petkova E., Stankov A., Mann J.J., Rosoklija G., Dwork A.J. (2016). Reduced GABA neuron density in auditory cerebral cortex of subjects with major depressive disorder. J. Chem. Neuroanat..

[170] Grieve S.M., Korgaonkar M.S., Koslow S.H., Gordon E., Williams L.M. (2013). Widespread reductions in gray matter volume in depression. NeuroImage Clin..

[171] Järnum H., Eskildsen S.F., Steffensen E.G., Lundbye-Christensen S., Simonsen C.W., Thomsen I.S., Fründ E.-T., Théberge J., Larsson E.-M. (2011). Longitudinal MRI study of cortical thickness, perfusion, and metabolite levels in major depressive disorder. Acta Psychiatr. Scand..

[172] Magalhães R., Barrière D.A., Novais A., Marques F., Marques P., Cerqueira J., Sousa J.C., Cachia A., Boumezbeur F., Bottlaender M. (2018). The dynamics of stress: A longitudinal MRI study of rat brain structure and connectome. Mol. Psychiatry.

[173] Maren S., Yap S.A., Goosens K.A. (2001). The amygdala is essential for the development of neuronal plasticity in the medial geniculate nucleus during auditory fear conditioning in rats. J. Neurosci..

[174] Gong Q., Wu Q., Scarpazza C., Lui S., Jia Z., Marquand A., Huang X., McGuire P., Mechelli A. (2011). Prognostic prediction of therapeutic response in depression using high-field MR imaging. Neuroimage.

[175] Zhao Y.J., Du M.Y., Huang X.Q., Lui S., Chen Z.Q., Liu J., Luo Y., Wang X.L., Kemp G.J., Gong Q.Y. (2014). Brain grey matter abnormalities in medication-free patients with major depressive disorder: A meta-analysis. Psychol. Med..

[176] Roozendaal B., McEwen B.S., Chattarji S. (2009). Stress, memory and the amygdala. Nat. Rev. Neurosci..

[177] Bylsma L.M., Morris B.H., Rottenberg J. (2008). A meta-analysis of emotional reactivity in major depressive disorder. Clin. Psychol. Rev..

[178] Hamilton J.P., Siemer M., Gotlib I.H. (2008). Amygdala volume in major depressive disorder: A meta-analysis of magnetic resonance imaging studies. Mol. Psychiatry.

[179] Sheline Y.I., Barch D.M., Donnelly J.M., Ollinger J.M., Snyder A.Z., Mintun M.A. (2001). Increased amygdala response to masked emotional faces in depressed subjects resolves with antidepressant treatment: An fMRI study. Biol. Psychiatry.

[180] Vyas A., Mitra R., Shankaranarayana Rao B.S., Chattarji S. (2002). Chronic stress induces contrasting patterns of dendritic remodeling in hippocampal and amygdaloid neurons. J. Neurosci..

[181] Vyas A., Pillai A.G., Chattarji S. (2004). Recovery after chronic stress fails to reverse amygdaloid neuronal hypertrophy and enhanced anxiety-like behavior. Neuroscience.

[182] Roozendaal B., McReynolds J.R., McGaugh J.L. (2004). The Basolateral Amygdala Interacts with the Medial Prefrontal Cortex in Regulating Glucocorticoid Effects on Working Memory Impairment. J. Neurosci..

[183] Wilson M.A., Grillo C.A., Fadel J.R., Reagan L.P. (2015). Stress as a one-armed bandit: Differential effects of stress paradigms on the morphology, neurochemistry and behavior in the rodent amygdala. Neurobiol. Stress.

[184] Réus G.Z., Fries G.R., Stertz L., Badawy M., Passos I.C., Barichello T., Kapczinski F., Quevedo J. (2015). The role of inflammation and microglial activation in the pathophysiology of psychiatric disorders. Neuroscience.

[185] Wohleb E.S., Franklin T., Iwata M., Duman R.S. (2016). Integrating neuroimmune systems in the neurobiology of depression. Nat. Rev. Neurosci..

[186] Bourgin J., Cachia A., Boumezbeur F., Djemaï B., Bottlaender M., Duchesnay E., Mériaux S., Jay T.M. (2015). Hyper-responsivity to stress in rats is associated with a large increase in amygdala volume. A 7T MRI study. Eur. Neuropsychopharmacol..

[187] Leuner B., Shors T.J. (2013). Stress, anxiety, and dendritic spines: What are the connections?. Neuroscience.

[188] Sibille E., Wang Y., Joeyen-Waldorf J., Gaiteri C., Surget A., Oh S., Belzung C., Tseng G.C., Lewis D.A. (2009). A molecular signature of depression in the amygdala. Am. J. Psychiatry.

[189] Savalli G., Diao W., Schulz S., Todtova K., Pollak D.D. (2015). Diurnal oscillation of Amygdala clock gene expression and loss of synchrony in a mouse model of depression. Int. J. Neuropsychopharmacol..

[190] Bora E., Harrison B.J., Davey C.G., Yücel M., Pantelis C. (2012). Meta-analysis of volumetric abnormalities in cortico-striatal-pallidal- thalamic circuits in major depressive disorder. Psychol. Med..

[191] Pizzagalli D.A., Holmes A.J., Dillon D.G., Goetz E.L., Birk J.L., Bogdan R., Dougherty D.D., Iosifescu D.V., Rauch S.L., Fava M. (2009). Reduced caudate and nucleus accumbens response to rewards in unmedicated individuals with major depressive disorder. Am. J. Psychiatry.

[192] Khundakar A., Morris C., Oakley A., Thomas A.J. (2011). Morphometric analysis of neuronal and glial cell pathology in the caudate nucleus in late-life depression. Am. J. Geriatr. Psychiatry.

[193] Hickie I., Ward P., Scott E., Haindl W., Walker B., Dixon J., Turner K. (1999). Neo-striatal rCBF correlates of psychomotor slowing in patients with major depression. Psychiatry Res. Neuroimaging.

[194] Delgado y Palacios R., Verhoye M., Henningsen K., Wiborg O., Van der Linden A. (2014). Diffusion Kurtosis Imaging and High-Resolution MRI Demonstrate Structural Aberrations of Caudate Putamen and Amygdala after Chronic Mild Stress. PLoS ONE.

[195] Hemanth Kumar B.S., Mishra S.K., Trivedi R., Singh S., Rana P., Khushu S. (2014). Demyelinating evidences in CMS rat model of depression: A DTI study at 7T. Neuroscience.

[196] Van Velzen L.S., Kelly S., Isaev D., Aleman A., Aftanas L.I., Bauer J., Baune B.T., Brak I.V., Carballedo A., Connolly C.G. (2019). White matter disturbances in major depressive disorder: A coordinated analysis across 20 international cohorts in the ENIGMA MDD working group. Mol. Psychiatry.

[197] Dillon D.G., Gonenc A., Belleau E., Pizzagalli D.A. (2018). Depression is associated with dimensional and categorical effects on white matter pathways. Depress. Anxiety.

[198] Bracht T., Horn H., Strik W., Federspiel A., Schnell S., Höfle O., Stegmayer K., Wiest R., Dierks T., Müller T.J. (2014). White matter microstructure alterations of the medial forebrain bundle in melancholic depression. J. Affect. Disord..

[199] Kamiya K., Okada N., Sawada K., Watanabe Y., Irie R., Hanaoka S., Suzuki Y., Koike S., Mori H., Kunimatsu A. (2018). Diffusional kurtosis imaging and white matter microstructure modeling in a clinical study of major depressive disorder. NMR Biomed..

[200] Fieremans E., Jensen J.H., Helpern J.A. (2011). White matter characterization with diffusional kurtosis imaging. Neuroimage.

[201] Hansen B., Jespersen S.N. (2017). Recent developments in fast kurtosis imaging. Front. Phys..

[202] Lawson R.P., Nord C.L., Seymour B., Thomas D.L., Dayan P., Pilling S., Roiser J.P. (2017). Disrupted habenula function in major depression. Mol. Psychiatry.

[203] Savitz J.B., Nugent A.C., Bogers W., Roiser J.P., Bain E.E., Neumeister A., Zarate C.A., Manji H.K., Cannon D.M., Marrett S. (2011). Habenula volume in bipolar disorder and major depressive disorder: A high-resolution magnetic resonance imaging study. Biol. Psychiatry.

[204] Li K., Zhou T., Liao L., Yang Z., Wong C., Henn F., Malinow R., Yates J.R., Hu H. (2013). βCaMKII in lateral habenula mediates core symptoms of depression. Science (80-.).

[205] Christensen T., Jensen L., Bouzinova E.V., Wiborg O. (2013). Molecular profiling of the lateral habenula in a rat model of depression. PLoS ONE.

[206] Shen X.F., Yuan H.B., Wang G.Q., Xue H., Liu Y.F., Zhang C.X. (2019). Role of DNA hypomethylation in lateral habenular nucleus in the development of depressive-like behavior in rats. J. Affect. Disord..

[207] Faron-Górecka A., Kuśmider M., Kolasa M., Zurawek D., Szafran-Pilch K., Gruca P., Pabian P., Solich J., Papp M., Dziedzicka-Wasylewska M. (2016). Chronic mild stress alters the somatostatin receptors in the rat brain. Psychopharmacology (Berlin).

[208] Ågren H., Lundqvist G. (1984). Low levels of somatostatin in human CSF mark depressive episodes. Psychoneuroendocrinology.

[209] Lin L.C., Sibille E. (2015). Somatostatin, neuronal vulnerability and behavioral emotionality. Mol. Psychiatry.

[210] Fee C., Banasr M., Sibille E. (2017). Somatostatin-Positive Gamma-Aminobutyric Acid Interneuron Deficits in Depression: Cortical Microcircuit and Therapeutic Perspectives. Biol. Psychiatry.

[211] Carlezon W.A., Thomas M.J. (2009). Biological substrates of reward and aversion: A nucleus accumbens activity hypothesis. Neuropharmacology.

[212] Bessa J.M., Morais M., Marques F., Pinto L., Palha J.A., Almeida O.F.X., Sousa N. (2013). Stress-induced anhedonia is associated with hypertrophy of medium spiny neurons of the nucleus accumbens. Transl. Psychiatry.

[213] Logan R.W., Edgar N., Gillman A.G., Hoffman D., Zhu X., McClung C.A. (2015). Chronic Stress Induces Brain Region-Specific Alterations of Molecular Rhythms that Correlate with Depression-like Behavior in Mice. Biol. Psychiatry.

[214] Ressler K.J., Nemeroff C.B. (2000). Role of serotonergic and noradrenergic systems in the pathophysiology of depression and anxiety disorders. Depress. Anxiety.

[215] Schildkraut J.J. (1965). The catecholamine hypothesis of affective disorders: A review of supporting evidence. Am. J. Psychiatry.

[216] Schildkraut J.J., Kety S.S. (1967). Biogenic amines and emotion. Science (80-. ).

[217] Massart R., Mongeau R., Lanfumey L. (2012). Beyond the monoaminergic hypothesis: Neuroplasticity and epigenetic changes in a transgenic mouse model of depression. Philos. Trans. R. Soc. B Biol. Sci..

[218] Zhang H., Li K., Chen H.S., Gao S.Q., Xia Z.X., Zhang J.T., Wang F., Chen J.G. (2018). Dorsal raphe projection inhibits the excitatory inputs on lateral habenula and alleviates depressive behaviors in rats. Brain Struct. Funct..

[219] Germain A., Kupfer D.J. (2008). Circadian rhythm disturbances in depression. Hum. Psychopharmacol. Clin. Exp..

[220] Bunney B.G., Li J.Z., Walsh D.M., Stein R., Vawter M.P., Cartagena P., Barchas J.D., Schatzberg A.F., Myers R.M., Watson S.J. (2015). Circadian dysregulation of clock genes: Clues to rapid treatments in major depressive disorder. Mol. Psychiatry.

[221] Bunney W.E., Bunney B.G. (2000). Molecular clock genes in man and lower animals: Possible implications for circadian abnormalities in depression. Neuropsychopharmacology.

[222] Kato M., Slack F.J. (2008). microRNAs: Small molecules with big roles -C. elegans to human cancer. Biol. Cell.

[223] Lopez J.P., Kos A., Turecki G. (2018). Major depression and its treatment: MicroRNAs as peripheral biomarkers of diagnosis and treatment response. Curr. Opin. Psychiatry.

[224] Ferrúa C.P., Giorgi R., da Rosa L.C., do Amaral C.C., Ghisleni G.C., Pinheiro R.T., Nedel F. (2019). MicroRNAs expressed in depression and their associated pathways: A systematic review and a bioinformatics analysis. J. Chem. Neuroanat..

[225] Fries G.R., Zhang W., Benevenuto D., Quevedo J. (2019). MicroRNAs in Major Depressive Disorder. Advances in Experimental Medicine and Biology.

[226] Cogswell J., Ward J., Taylor I., Waters M., Shi Y., Cannon B., Kelnar K., Kemppainen J., Brown D., Chen C. (2008). Identification of miRNA Changes in Alzheimer’s.pdf. J. Alzheimer’s Dis..

[227] Beveridge N.J., Cairns M.J. (2012). MicroRNA dysregulation in schizophrenia. Neurobiol. Dis..

[228] Burgos K., Malenica I., Metpally R., Courtright A., Rakela B., Beach T., Shill H., Adler C., Sabbagh M., Villa S. (2014). Profiles of extracellular miRNA in cerebrospinal fluid and serum from patients with Alzheimer’s and Parkinson’s diseases correlate with disease status and features of pathology. PLoS ONE.

[229] Moreau M.P., Bruse S.E., David-Rus R., Buyske S., Brzustowicz L.M. (2011). Altered MicroRNA expression profiles in postmortem brain samples from individuals with schizophrenia and bipolar disorder. Biol. Psychiatry.

[230] Chiang H.R., Schoenfeld L.W., Ruby J.G., Auyeung V.C., Spies N., Baek D., Johnston W.K., Russ C., Luo S., Babiarz J.E. (2010). Mammalian microRNAs: Experimental evaluation of novel and previously annotated genes. Genes Dev..

[231] Wheeler B.M., Heimberg A.M., Moy V.N., Sperling E.A., Holstein T.W., Heber S., Peterson K.J. (2009). The deep evolution of metazoan microRNAs. Evol. Dev..

[232] Meerson A., Cacheaux L., Goosens K.A., Sapolsky R.M., Soreq H., Kaufer D. (2010). Changes in brain MicroRNAs contribute to cholinergic stress reactions. J. Mol. Neurosci..

[233] Rinaldi A., Vincenti S., De Vito F., Bozzoni I., Oliverio A., Presutti C., Fragapane P., Mele A. (2010). Stress induces region specific alterations in microRNAs expression in mice. Behav. Brain Res..

[234] Zhou M., Wang M., Wang X., Liu K., Wan Y.Q., Li M., Liu L., Zhang C. (2018). Abnormal Expression of MicroRNAs Induced by Chronic Unpredictable Mild Stress in Rat Hippocampal Tissues. Mol. Neurobiol..

[235] Buran İ., Etem E.Ö., Tektemur A., Elyas H. (2017). Treatment with TREK1 and TRPC3/6 ion channel inhibitors upregulates microRNA expression in a mouse model of chronic mild stress. Neurosci. Lett..

[236] Dwivedi Y. (2011). Evidence demonstrating role of microRNAs in the etiopathology of major depression. J. Chem. Neuroanat..

[237] Bocchio-Chiavetto L., Maffioletti E., Bettinsoli P., Giovannini C., Bignotti S., Tardito D., Corrada D., Milanesi L., Gennarelli M. (2013). Blood microRNA changes in depressed patients during antidepressant treatment. Eur. Neuropsychopharmacol..

[238] Zurawek D., Kusmider M., Faron-Gorecka A., Gruca P., Pabian P., Solich J., Kolasa M., Papp M., Dziedzicka-Wasylewska M. (2017). Reciprocal MicroRNA Expression in Mesocortical Circuit and Its Interplay with Serotonin Transporter Define Resilient Rats in the Chronic Mild Stress. Mol. Neurobiol..

[239] Zurawek D., Kusmider M., Faron-Gorecka A., Gruca P., Pabian P., Kolasa M., Solich J., Szafran-Pilch K., Papp M., Dziedzicka-Wasylewska M. (2016). Time-dependent miR-16 serum fluctuations together with reciprocal changes in the expression level of miR-16 in mesocortical circuit contribute to stress resilient phenotype in chronic mild stress - An animal model of depression. Eur. Neuropsychopharmacol..

[240] Baudry A., Mouillet-Richard S., Schneider B., Launay J.M., Kellermann O. (2010). MiR-16 targets the serotonin transporter: A new facet for adaptive responses to antidepressants. Science (80-.).

[241] Higuchi F., Uchida S., Yamagata H., Abe-Higuchi N., Hobara T., Hara K., Kobayashi A., Shintaku T., Itoh Y., Suzuki T. (2016). Hippocampal microRNA-124 enhances chronic stress resilience in mice. J. Neurosci..

[242] Yu H., Fan C., Yang L., Yu S., Song Q., Wang P., Mao X. (2018). Ginsenoside Rg1 Prevents Chronic Stress-Induced Depression-Like Behaviors and Neuronal Structural Plasticity in Rats. Cell. Physiol. Biochem..

[243] Bahi A., Chandrasekar V., Dreyer J.L. (2014). Selective lentiviral-mediated suppression of microRNA124a in the hippocampus evokes antidepressants-like effects in rats. Psychoneuroendocrinology.

[244] Schratt G.M., Tuebing F., Nigh E.A., Kane C.G., Sabatini M.E., Kiebler M., Greenberg M.E. (2006). A brain-specific microRNA regulates dendritic spine development. Nature.

